# Supplementary material for: Development and Biological Evaluation of the First Highly Potent and Specific Benzamide-Based Radiotracer [18F]BA3 for Imaging of Histone Deacetylases 1 and 2 in Brain
Source: Pharmaceuticals (Basel). 2022 Mar 8;15(3):324. doi: 10.3390/ph15030324 (PMC8950173; doi:10.3390/ph15030324)
Supplement: Supplementary file 1 [file pharmaceuticals-15-00324-s001.zip › pharmaceuticals-1602179-supplementary.pdf]

pharmaceuticals

MDPI

Supplementary Materials

Development and biological evaluation of the first highly potent and selective benzamide-based radiotracer [^{18}F]BA3 for imaging of histone deacetylases 1 and 2 in brain

Oliver Clauß, Linda Schäker-Hübner, Barbara Wenzel, Magali Toussaint, Winnie Deuther-Conrad, Daniel Gündel, Rodrigo Teodoro, Sladjana Dukić-Stefanović, Friedrich-Alexander Ludwig, Klaus Kopka, Peter Brust, Finn K. Hansen and Matthias Scheunemann

Figure S1: Scheme of the synthesis module TRACERlab FX2 N for the radiosynthesis of [^{18}F]**BA3**

Figure S2: Representative (**A**) UV- and (**B**) radio-RP-HPLC chromatograms of formulated [^{18}F]**BA3**

Figure S3: Representative radio-MLC chromatograms of *in vivo* metabolism studies

Figure S4: Baseline time-activity curves (TACs) of CD-1 mice brain region after injection of [^{18}F]**BA3**

Figure S5: Biodistribution of [^{18}F]**BA3** at different time points derived from PET imaging

Table S1: Tissue biodistribution of radioactivity at different time point after i.v. injection of [^{18}F]**BA3** in CD-1 mice

Figure S6: Representative maximal intensity projection map of [^{18}F]**BA3**

Figure S7 - Figure S46: ^{1}H-, ^{13}C- and ^{19}F-NMR spectra and LC-MS chromatograms for compounds **BA1-BA10**

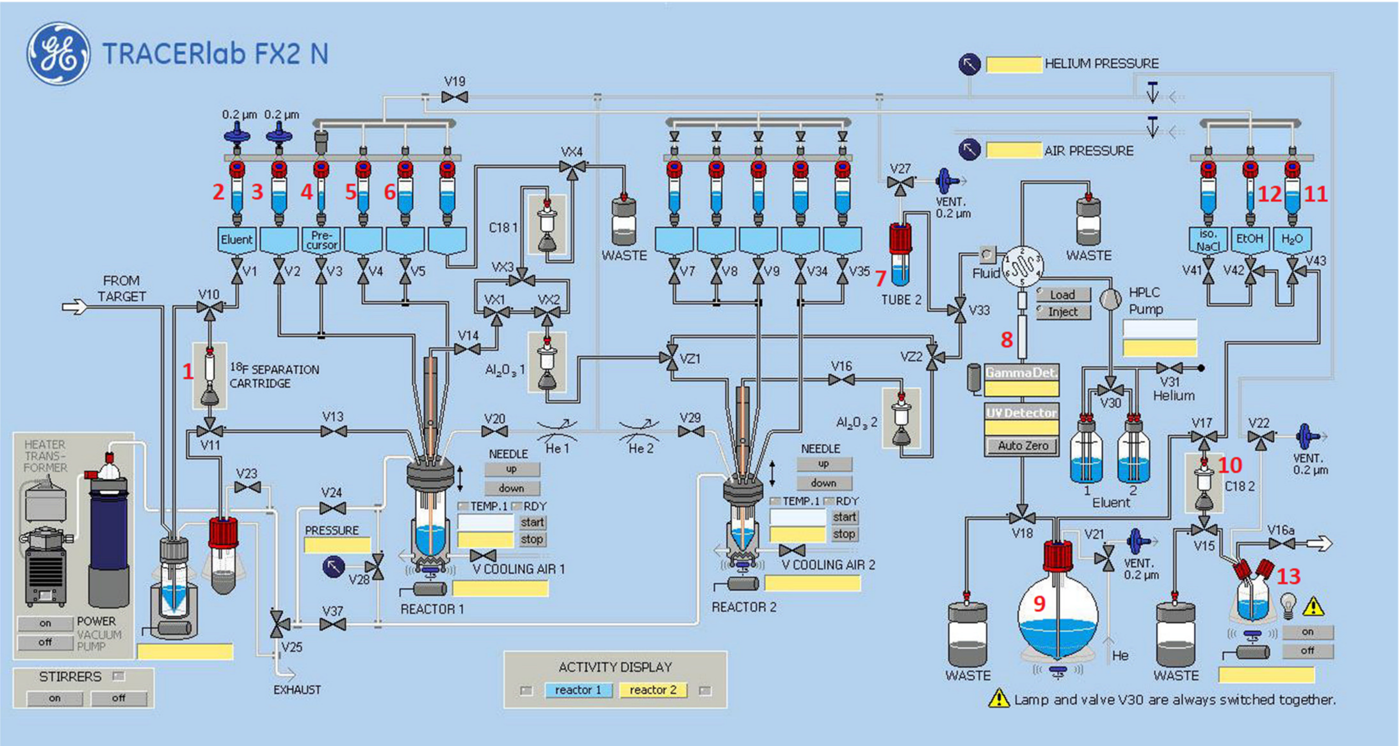

Figure S1. Scheme of the synthesis module TRACERlab FX2 N for the radiosynthesis of [^{18}F]**BA3**. (**1**) Sep-Pak® Accell QMA light cartridge, (**2**) 150 µL TBAHCO$_3$ (0.075 M) in 300 µL H_2O and 600 µL MeCN, (**3**) 2 mL MeCN, (**4**) precursor (4 mg of **9** in 800 µL MeCN), (**5**) 800 µL 2M HCl_{aq}, (**6**) 1.6 mL 1M $NaHCO_{3,aq}$ and 1.8 mL 100mM phosphate buffer (pH

= 6), (**7**) injection vial, (**8**) Reprosil-Pur C18-AQ (40% ACN/20mM NH_4OAc_{aq}, flow 4.0 mL/min), (**9**) 40 mL water, (**10**) Sep-Pak® C18 light, (**11**) 2 mL H_2O, (**12**) 1.2 mL EtOH, (**13**) product vial.

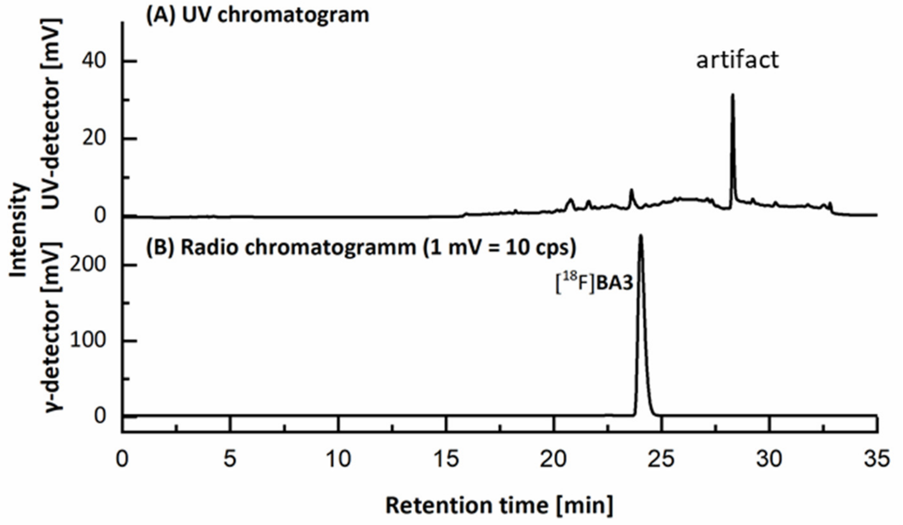

Figure S2. Representative (**A**) UV- and (**B**) radio-RP-HPLC chromatograms of formulated [^{18}F]**BA3** (ReproSil-Pur 120 C18-AQ column (250 x 4.6 mm, 5 µm), Gradient MeCN/20 mM $NH_4OAc_{aq.}$ (see quality control), flow rate: 1 mL/min).

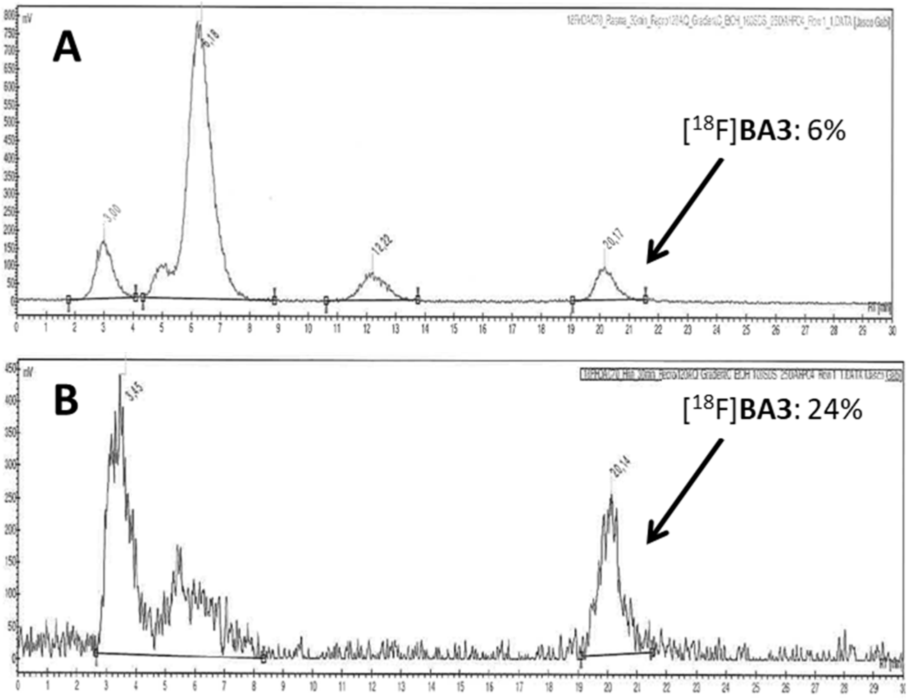

Figure S3. Representative radio-MLC chromatograms of *in vivo* metabolism studies from plasma (**A**) and brain (**B**) samples obtained 30 min p.i. of [^{18}F]**BA3** in a CD-1 mouse (gamma-detection via radio-RP-HPLC: ReproSil-Pur 120 C18-AQ column (250 x 4.6 mm, 10 µm + 10 mm pre-column), Gradient EtOH/100 mM SDS_{aq}/25 mM $(NH_4)_2HPO_4$ (see section 3.5.1.), flow: 1 mL/min).

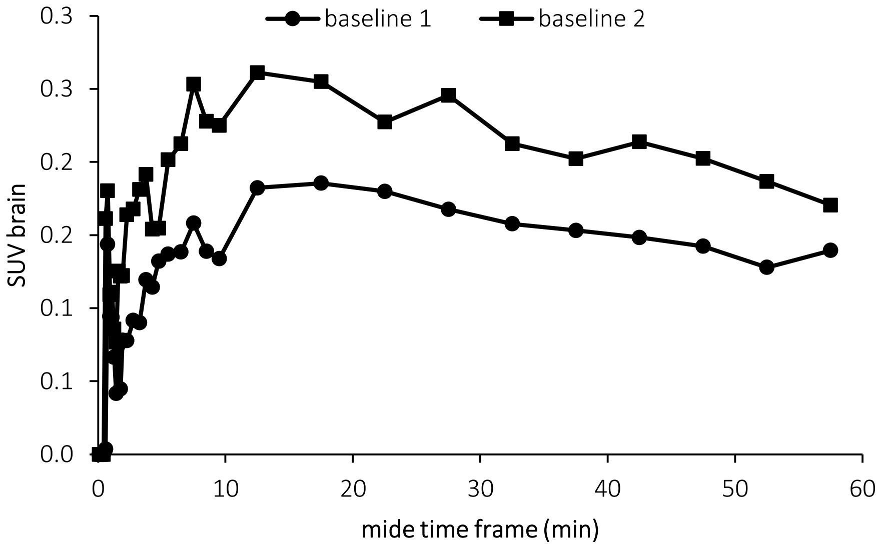

Figure S4. Baseline time-activity curves (TACs) of CD-1 mice brain region after injection of [^{18}F]**BA3** (n = 2).

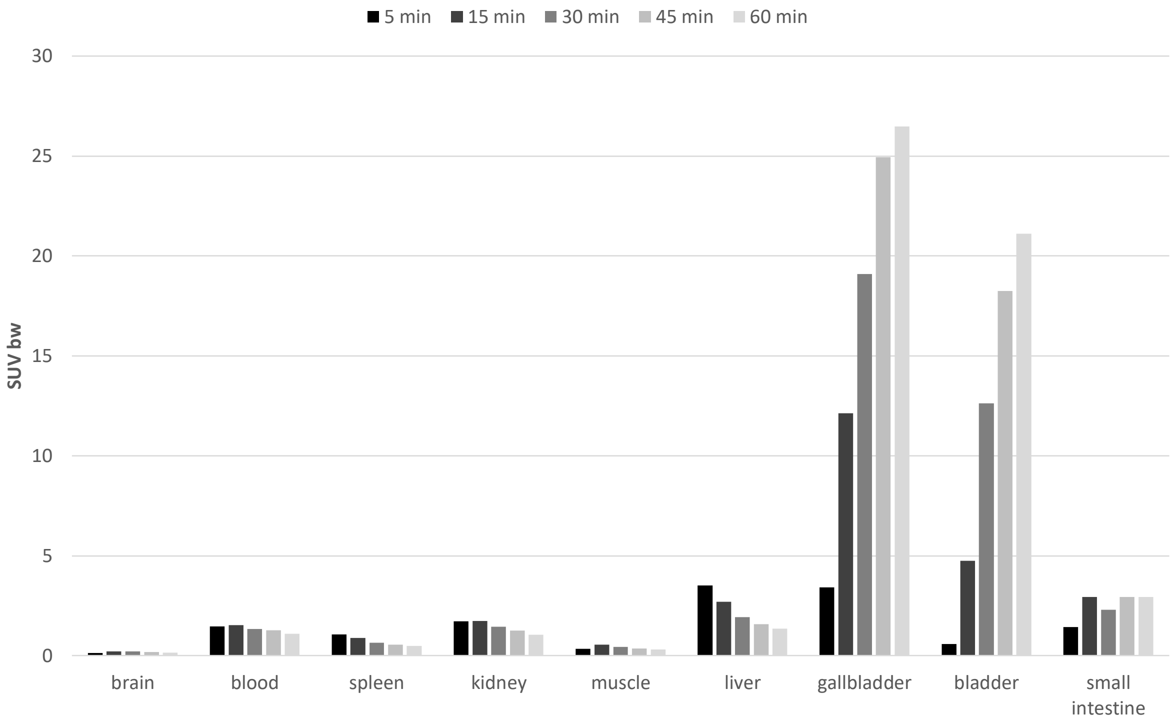

Figure S5. Biodistribution of [^{18}F]**BA3** at different time points derived from PET imaging uncorrected for metabolites (n = 2, SUV_{mean})

Table S1. Tissue biodistribution of radioactivity at different time point after i.v. injection of [^{18}F]**BA3** in CD-1 mice based on PET data uncorrected for metabolites (n = 2).

	**Uptake (SUV)**				
	**5 min**	**15 min**	**30 min**	**45 min**	**60 min**
**brain**	0.1 / 0.2	0.2 / 0.3	0.2 / 0.3	0.2 / 0.2	0.1 / 0.2
**blood**	1.7 / 1.2	1.8 / 1.2	1.6 / 1.0	1.6 / 0.9	1.4 / 0.9
**spleen**	1.2 / 0.9	1.0 / 0.8	0.7 / 0.6	0.6 / 0.5	0.5 / 0.5

**kidney**	1.7 / 1.7	1.7 / 1.8	1.4 / 1.6	1.1 / 1.4	0.9 / 1.2
**muscle**	0.3 / 0.4	0.6 / 0.6	0.5 / 0.4	0.4 / 0.4	0.3 / 0.3
**liver**	4.1 / 3.0	3.1 / 2.4	2.2 / 1.7	1.8 / 1.4	1.6 / 1.2
**gallbladder**	5.0 / 1.9	19.1 / 5.2	23.1 / 15.1	26.3 / 23.5	21.3 / 31.6
**bladder**	0.7 / 0.5	5.9 / 3.6	13.4 / 11.8	19.0 / 17.5	19.3 / 23.0
**small intestine**	1.7 / 1.2	3.7 / 2.2	2.4 / 2.2	2.5 / 3.4	3.0 / 2.9

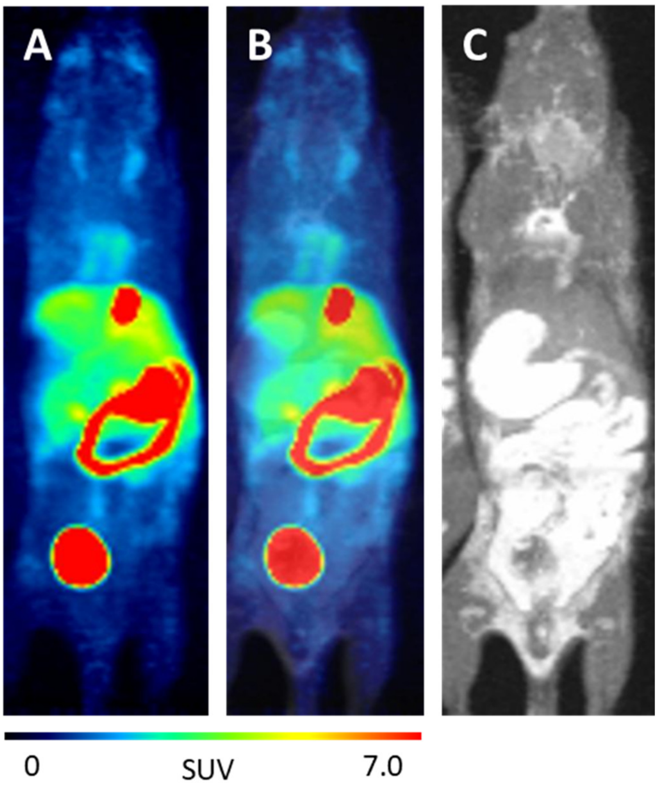

Figure S6. Representative maximal intensity projection map of [^{18}F]**BA3** of (**A**) PET modality or (**B**) merged PET and MR modalities and (**C**) MR modality.

N-[2-amino-5-(thiophen-2-yl)phenyl]-4-[(2-fluoropropanamido)methyl]benzamide (BA1)

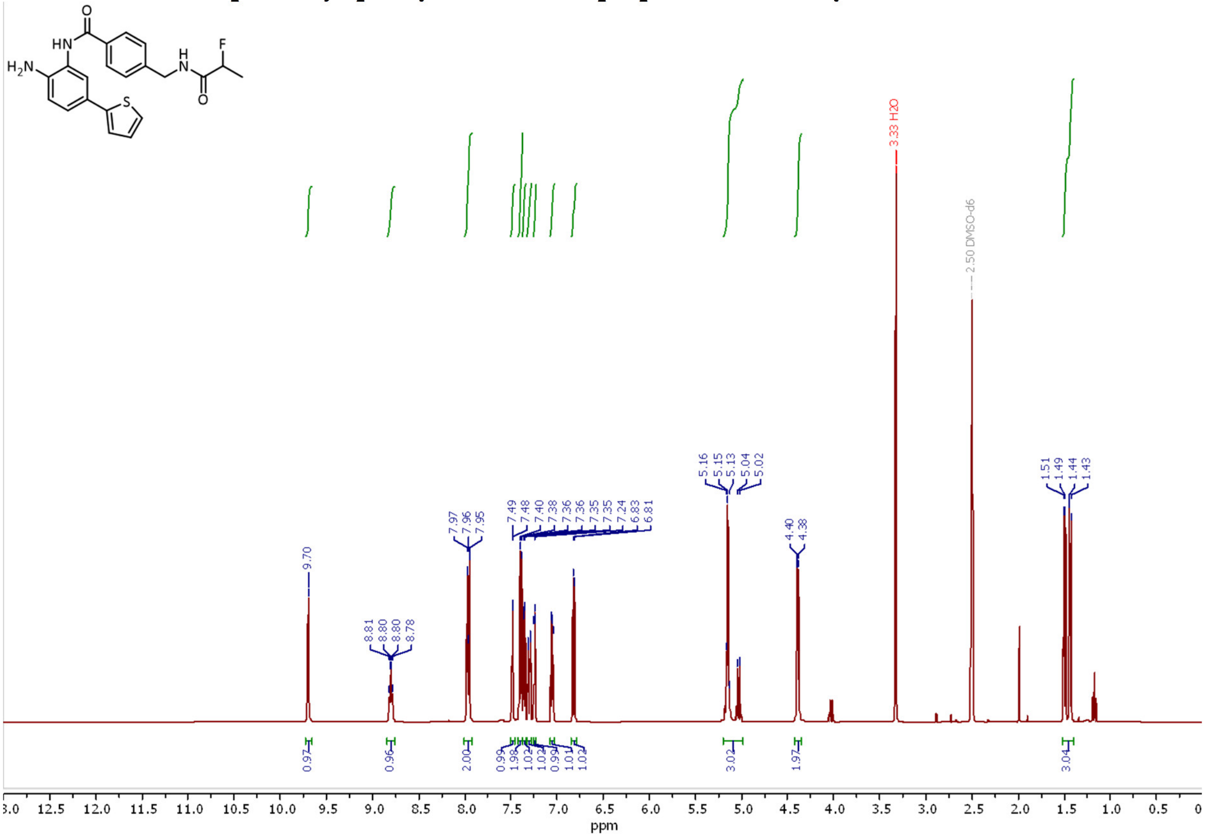

Figure S7. ^{1}H-NMR of **BA1**

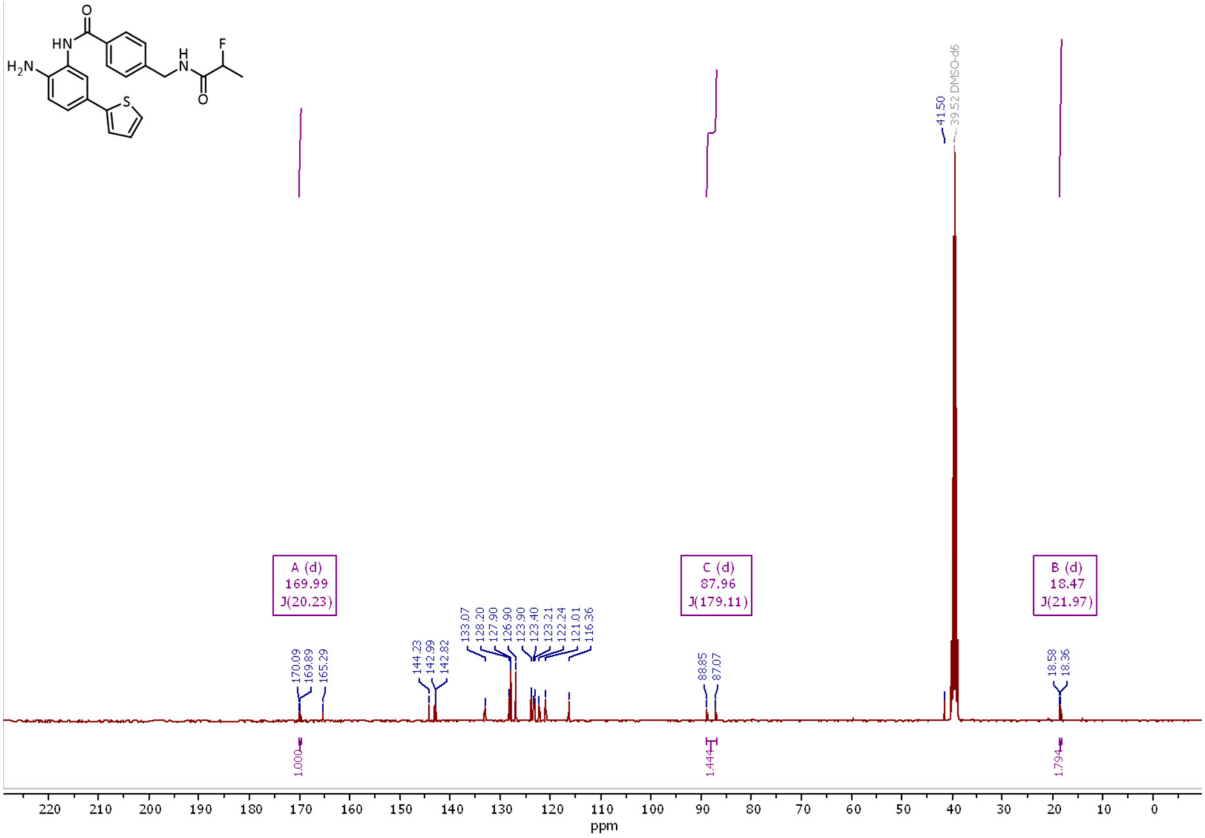

Figure S8. ^{13}C-NMR of **BA1**

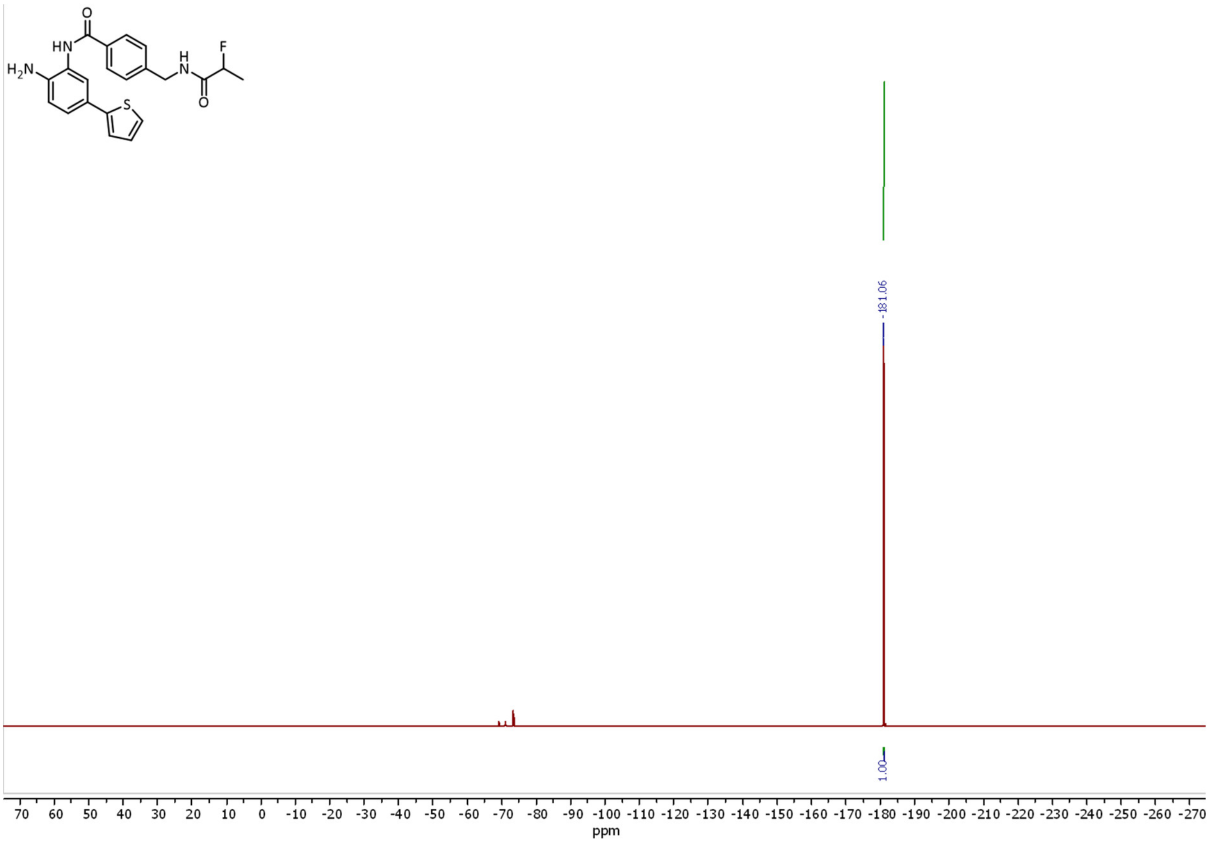

Figure S9. ^{19}F-NMR of **BA1**

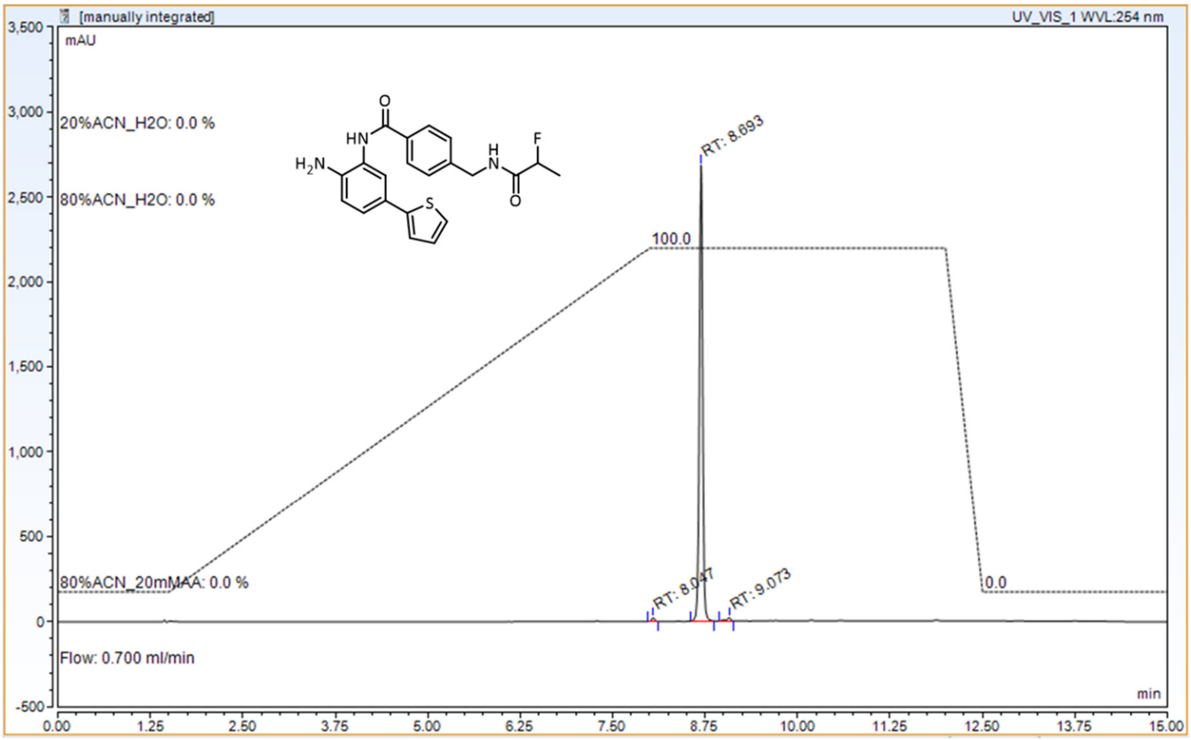

Figure S10. LC-MS chromatogram of **BA1**

N-[2-amino-5-(thiophen-2-yl)phenyl]-4-(2-fluoropropanamido)benzamide (BA2)

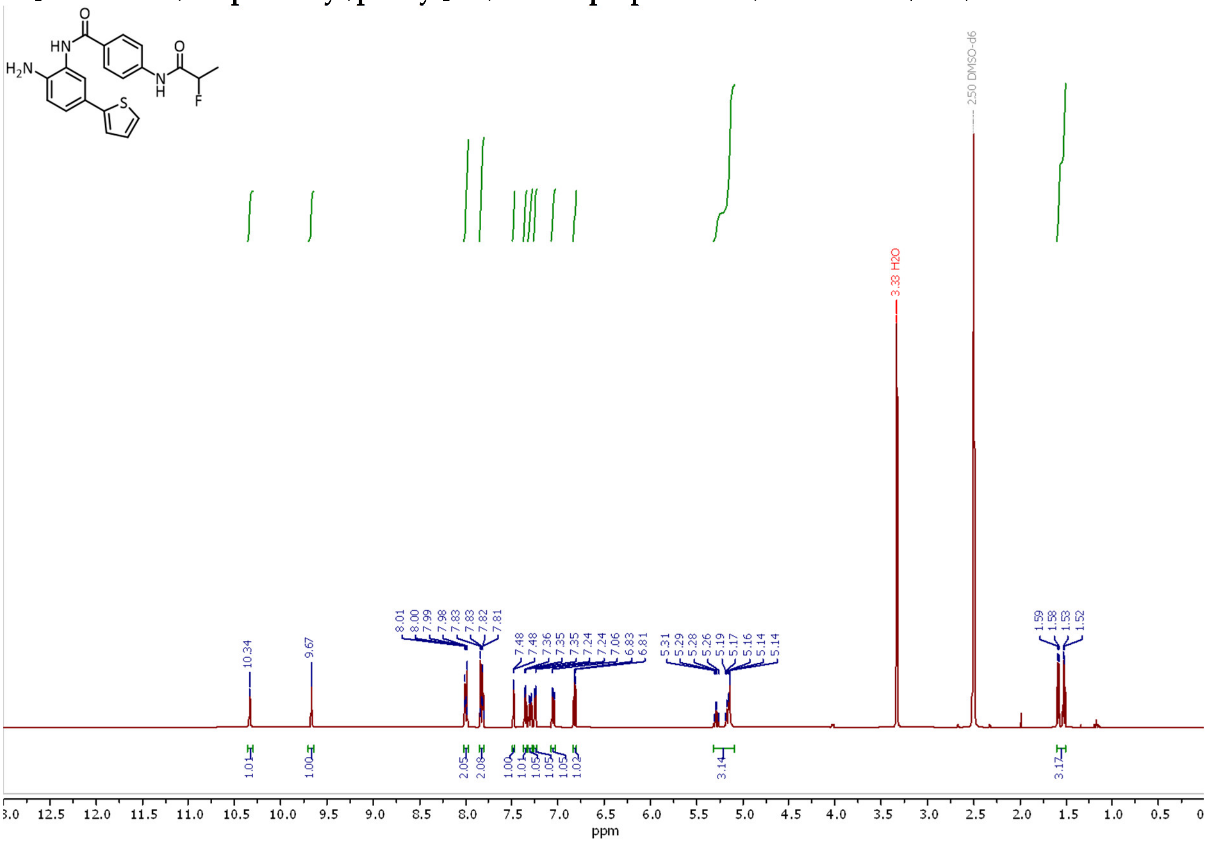

Figure S11. ^{1}H-NMR of **BA2**

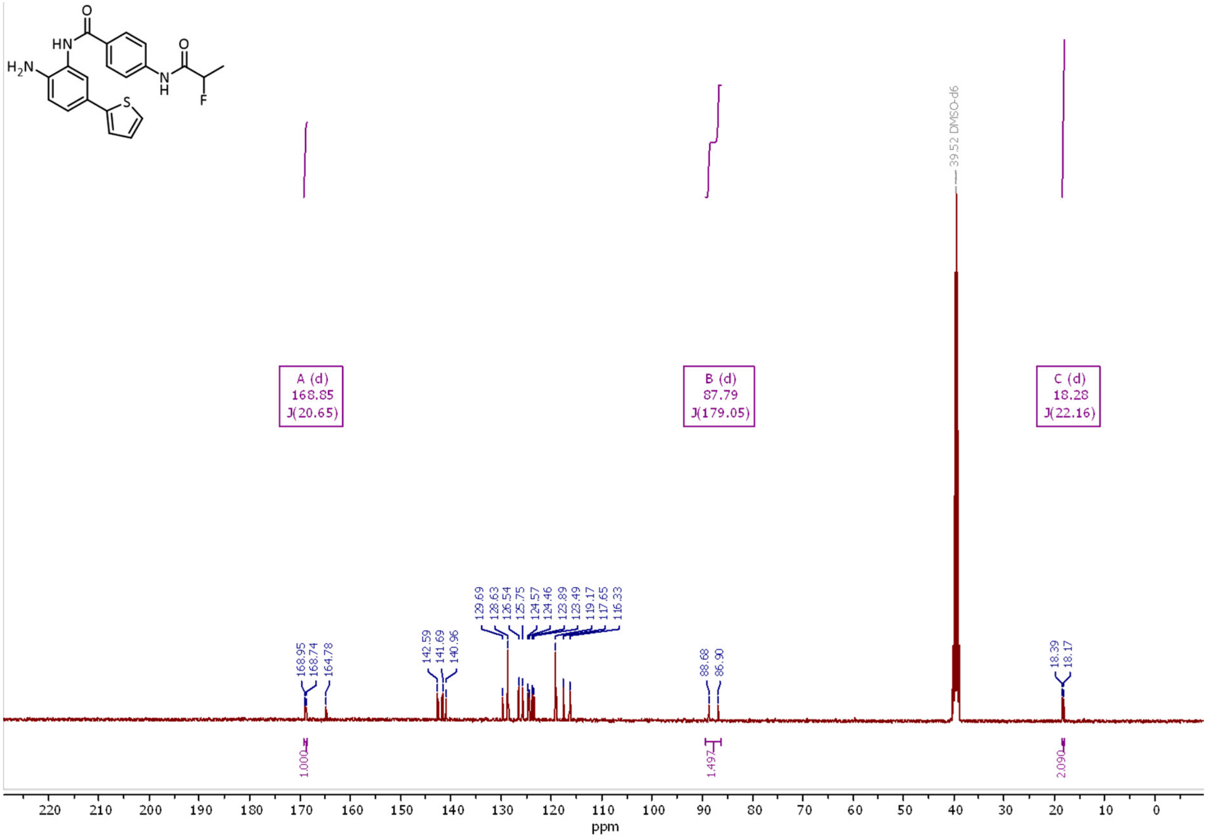

Figure S12. ^{13}C-NMR of **BA2**

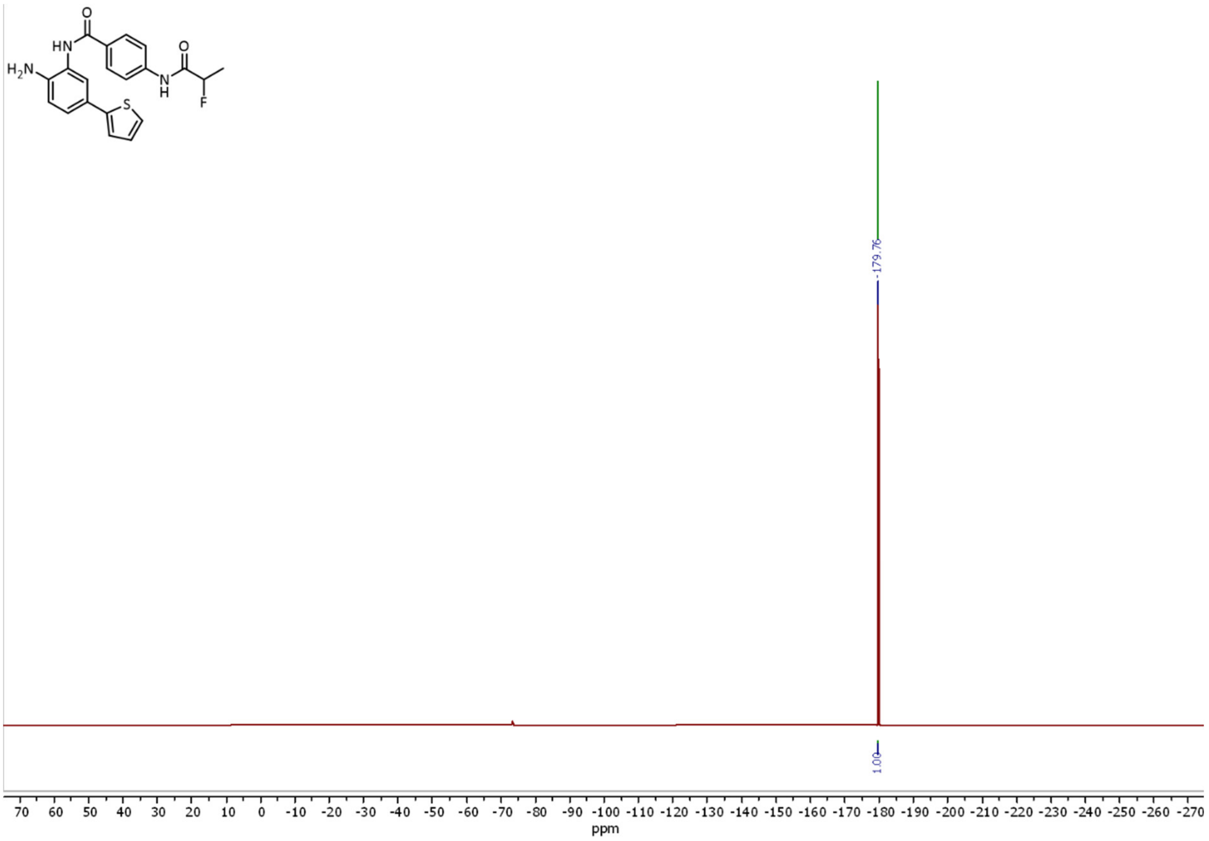

Figure S13. ^{19}F-NMR of **BA2**

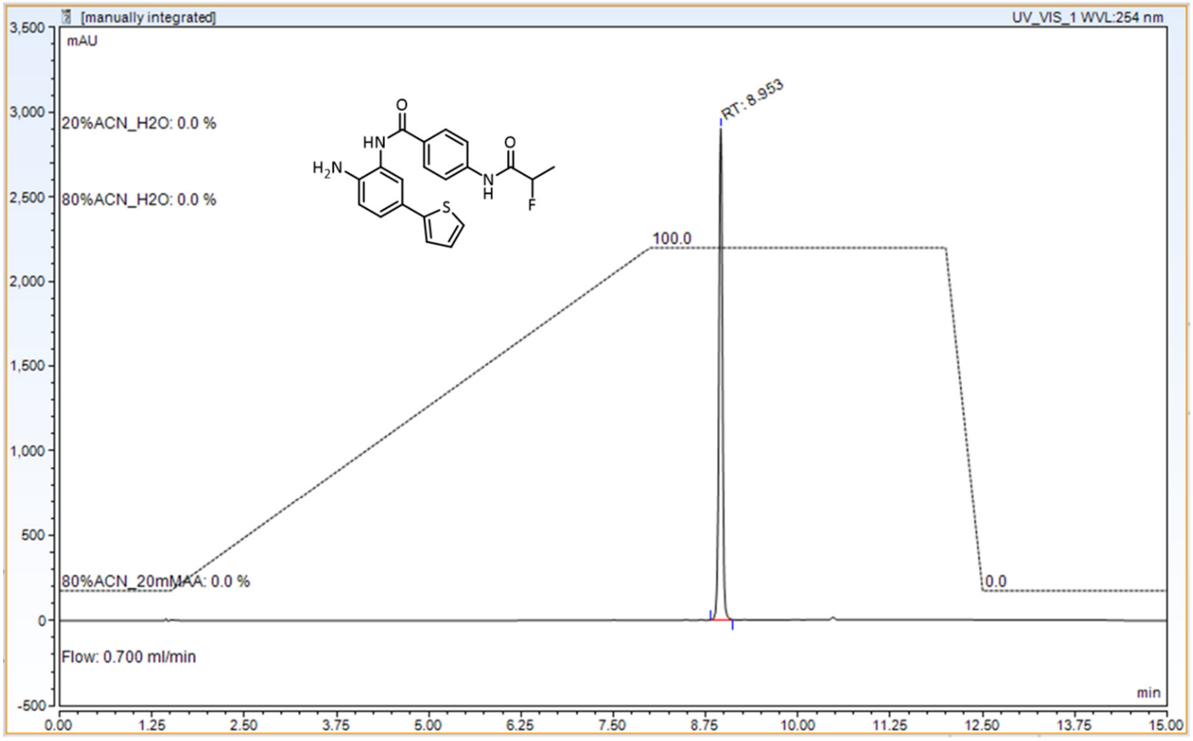

Figure S14. LC-MS chromatogram of **BA2**

***N*-[2-amino-5-(thiophen-3-yl)phenyl]-4-[(2-fluoropropanamido)methyl]benzamide (BA3)**

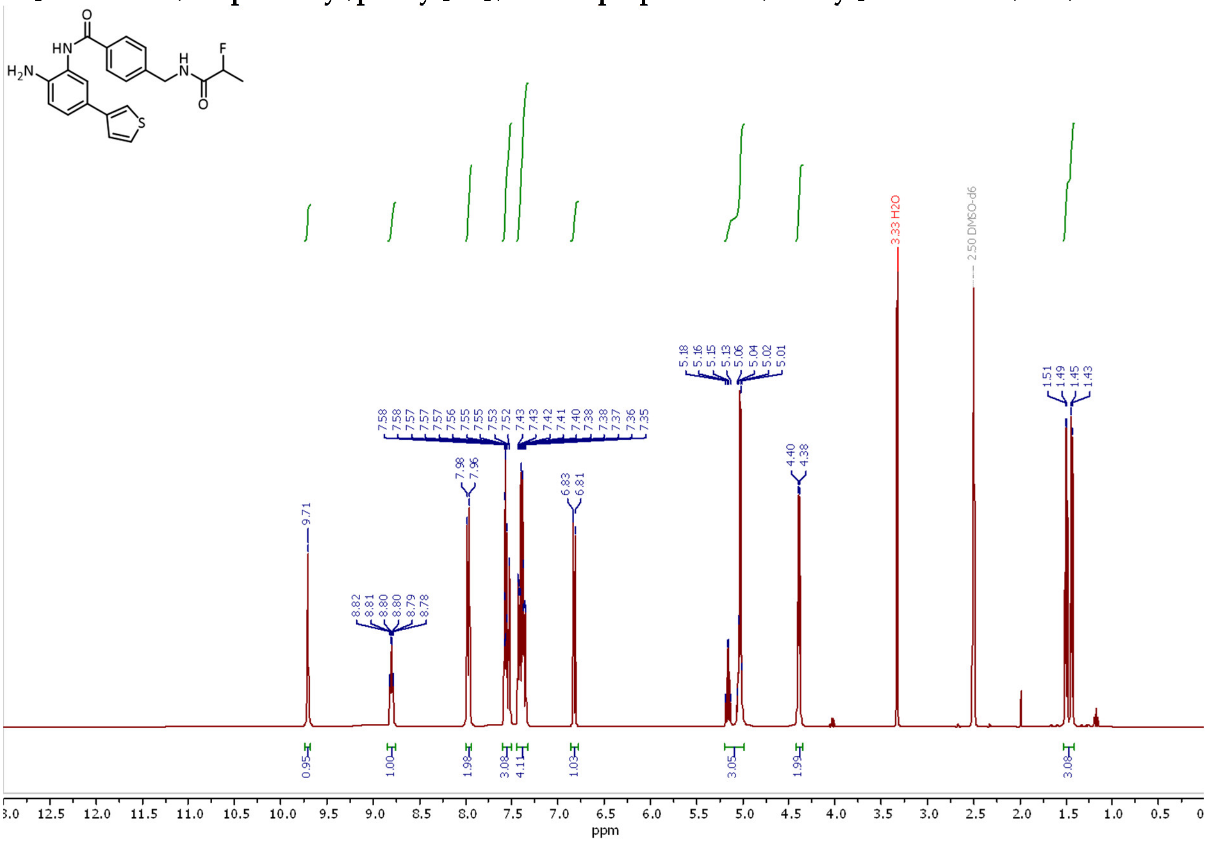

Figure S15. ^{1}H-NMR of **BA3**

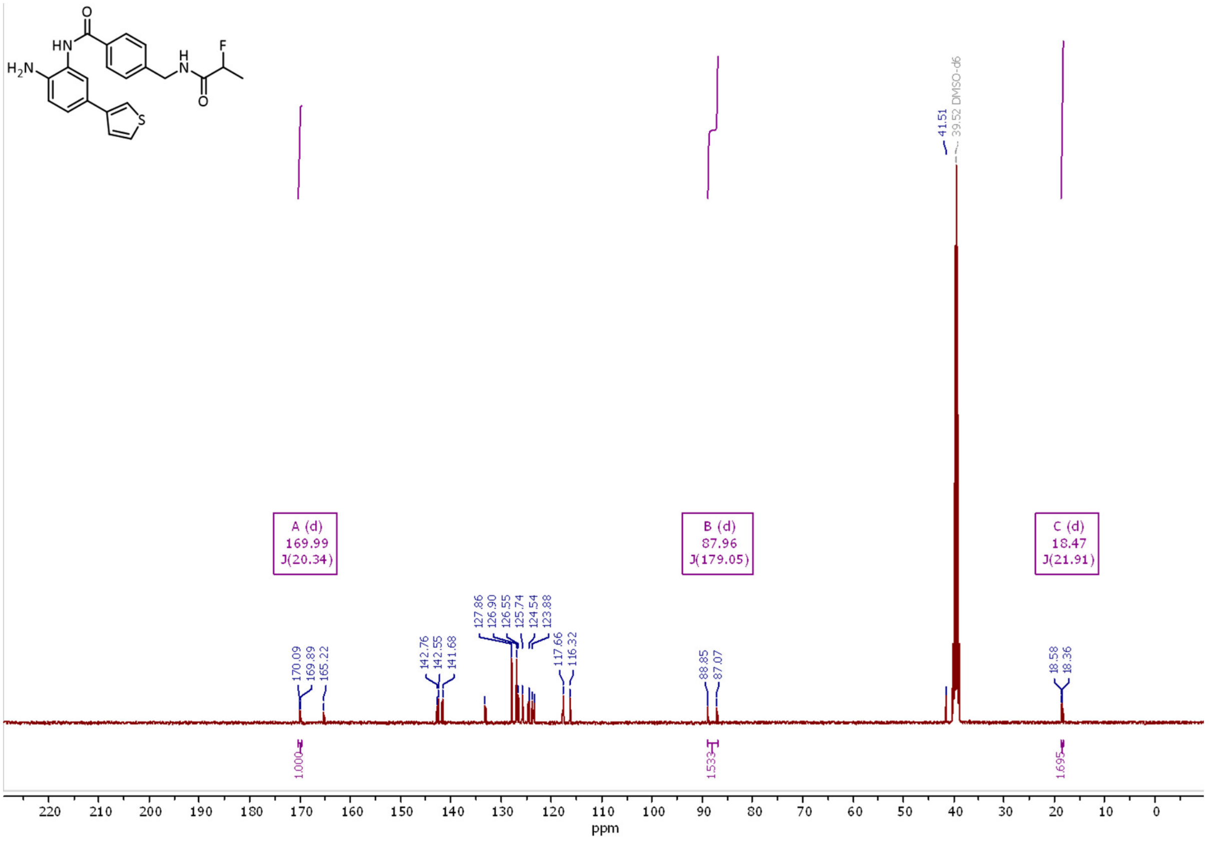

Figure S16. ^{13}C-NMR of **BA3**

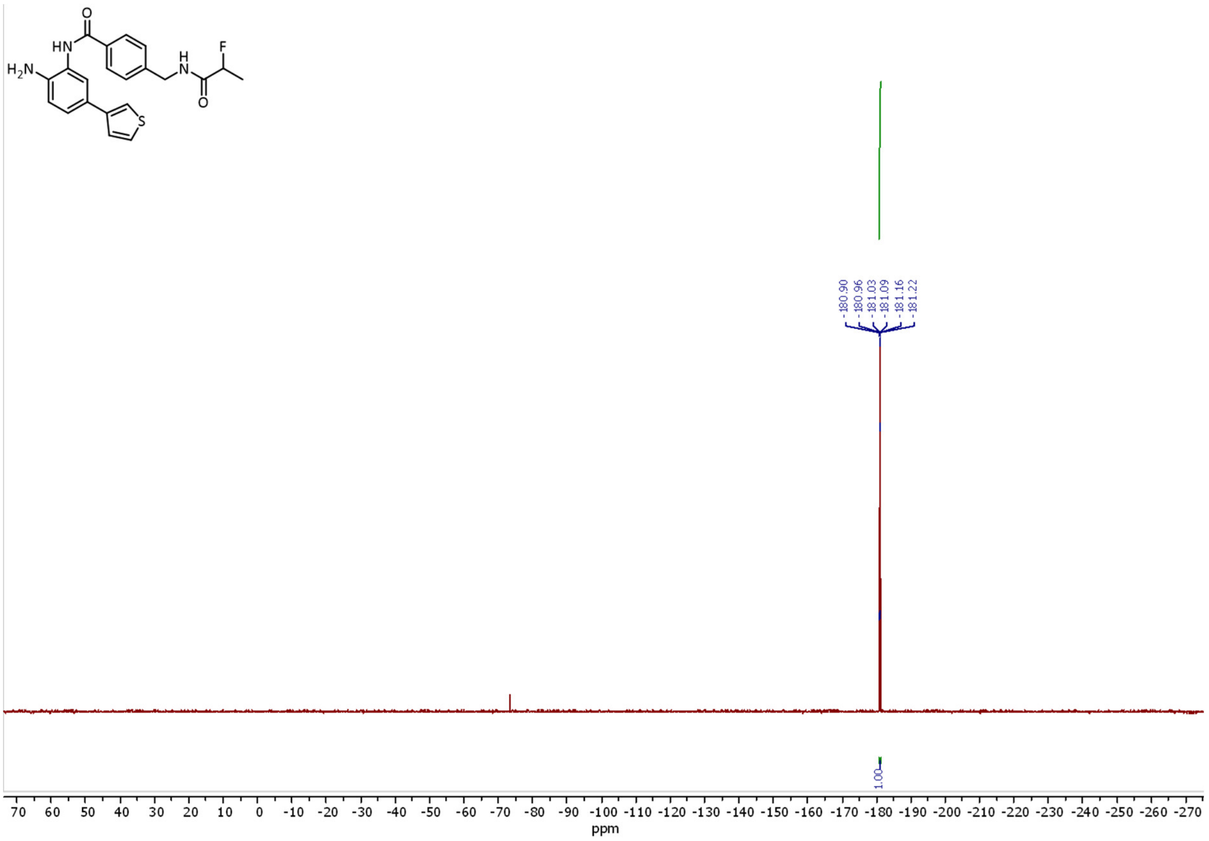

Figure S17. ^{19}F-NMR of **BA3**

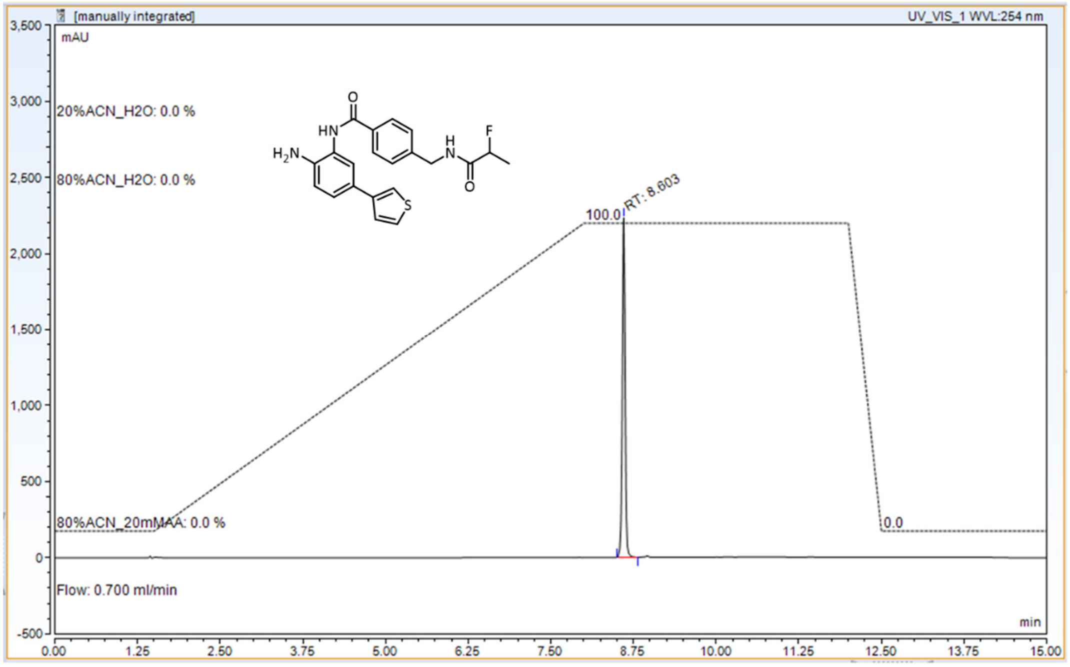

Figure S18. LC-MS chromatogram of **BA3**

N-[2-amino-5-(thiophen-3-yl)phenyl]-4-(2-fluoropropanamido)benzamide (BA4)

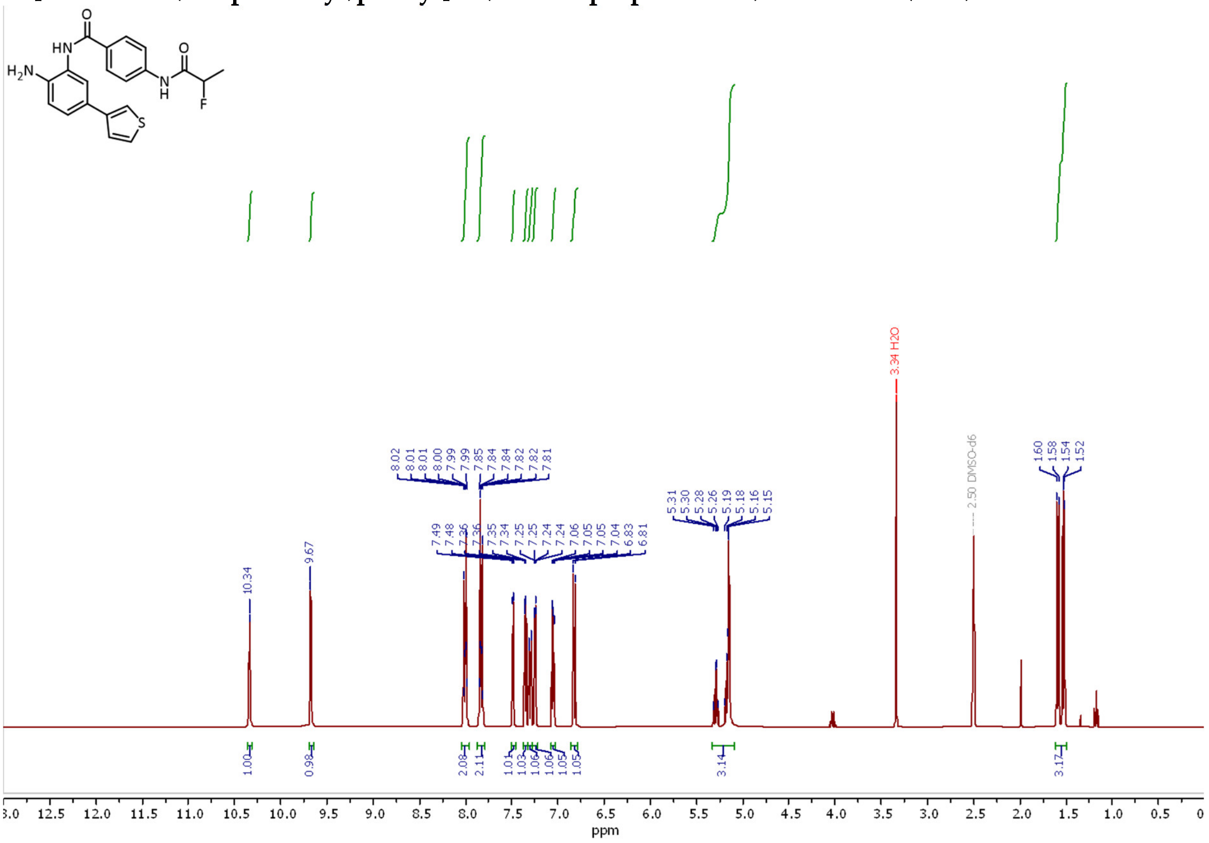

Figure S19. ^{1}H-NMR of **BA4**

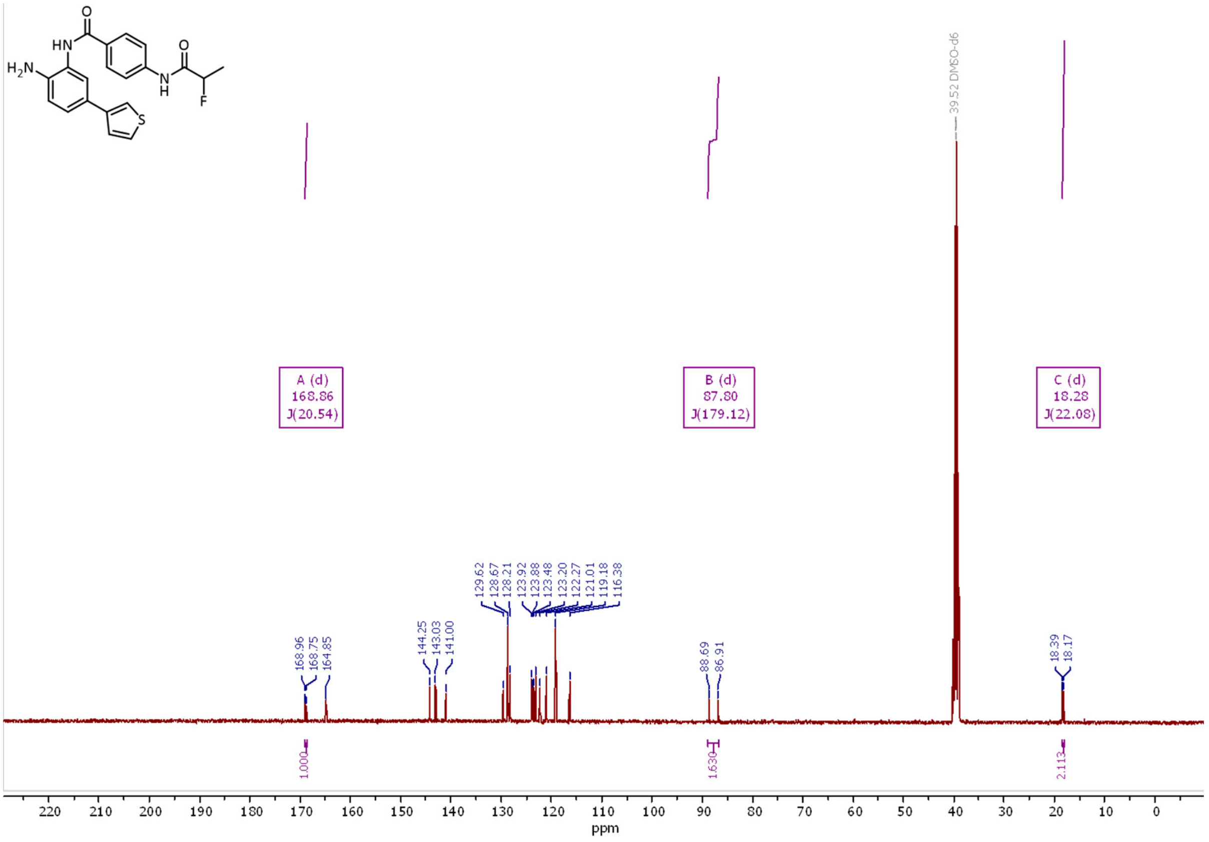

Figure S20. ^{13}C-NMR of **BA4**

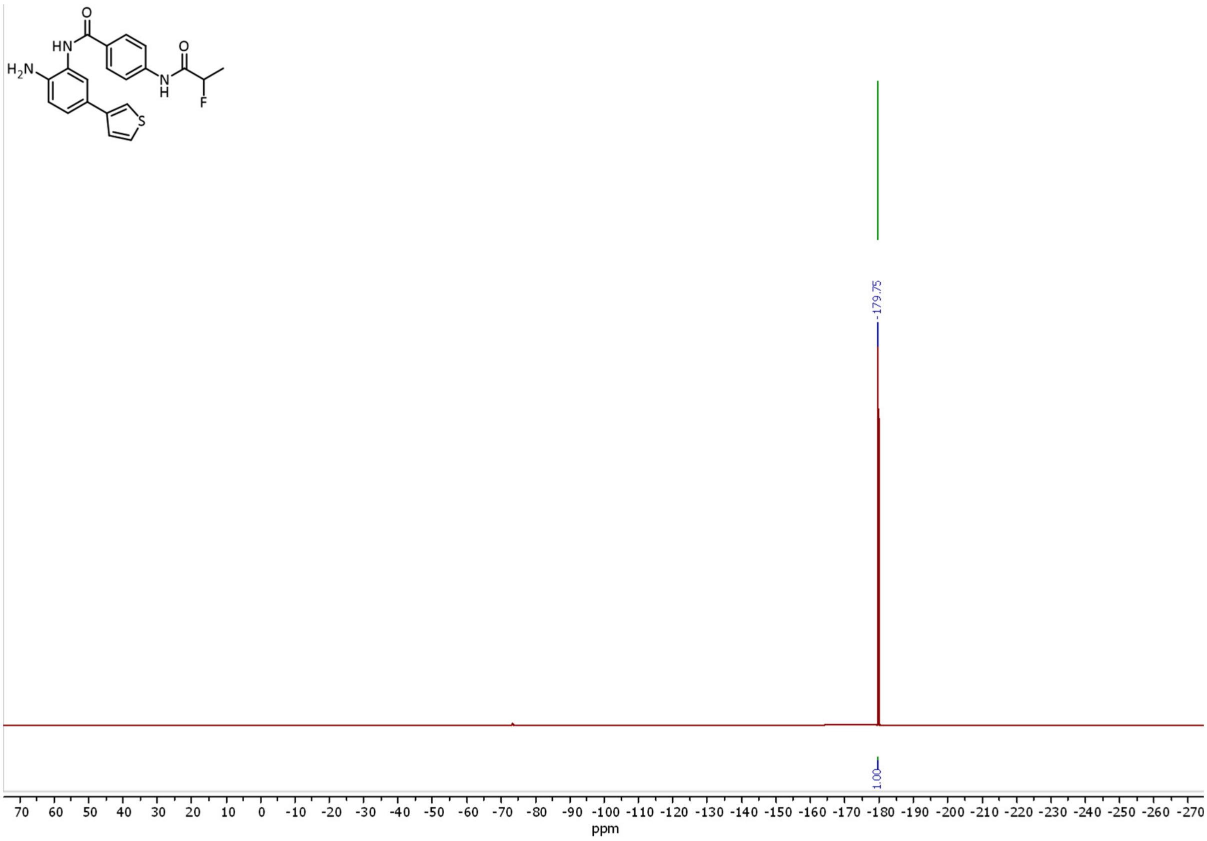

Figure S21. ^{19}F-NMR of **BA4**

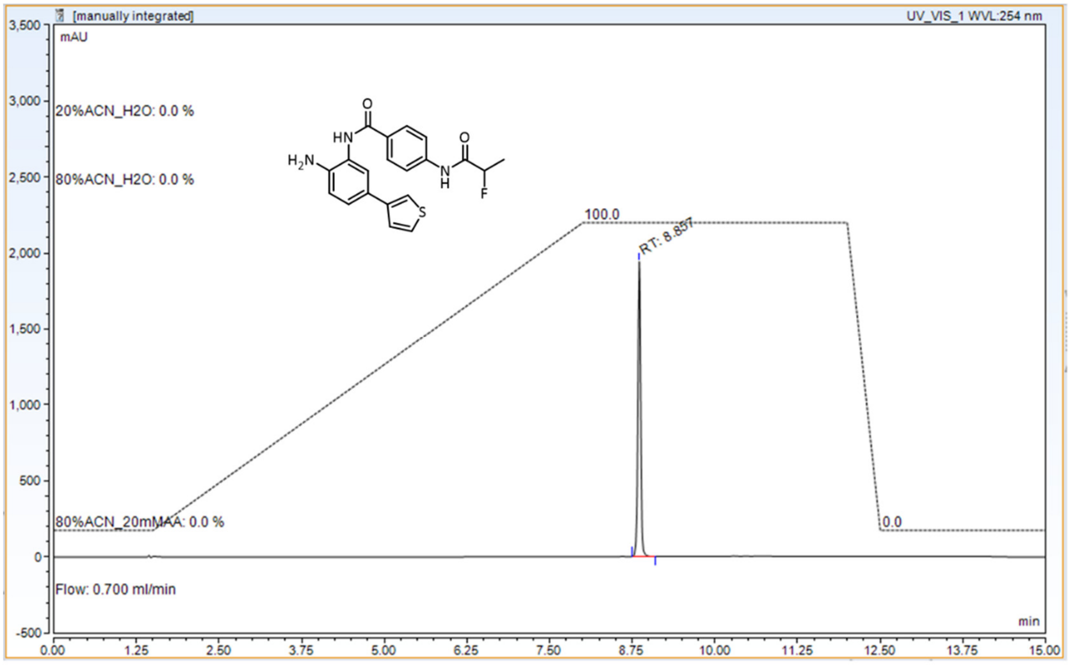

Figure S22. LC-MS chromatogram of **BA4**

N-(4-amino-4'-fluoro-[1,1'-biphenyl]-3-yl)-4-[(2-fluoropropanamido)methyl]benzamide (BA5)

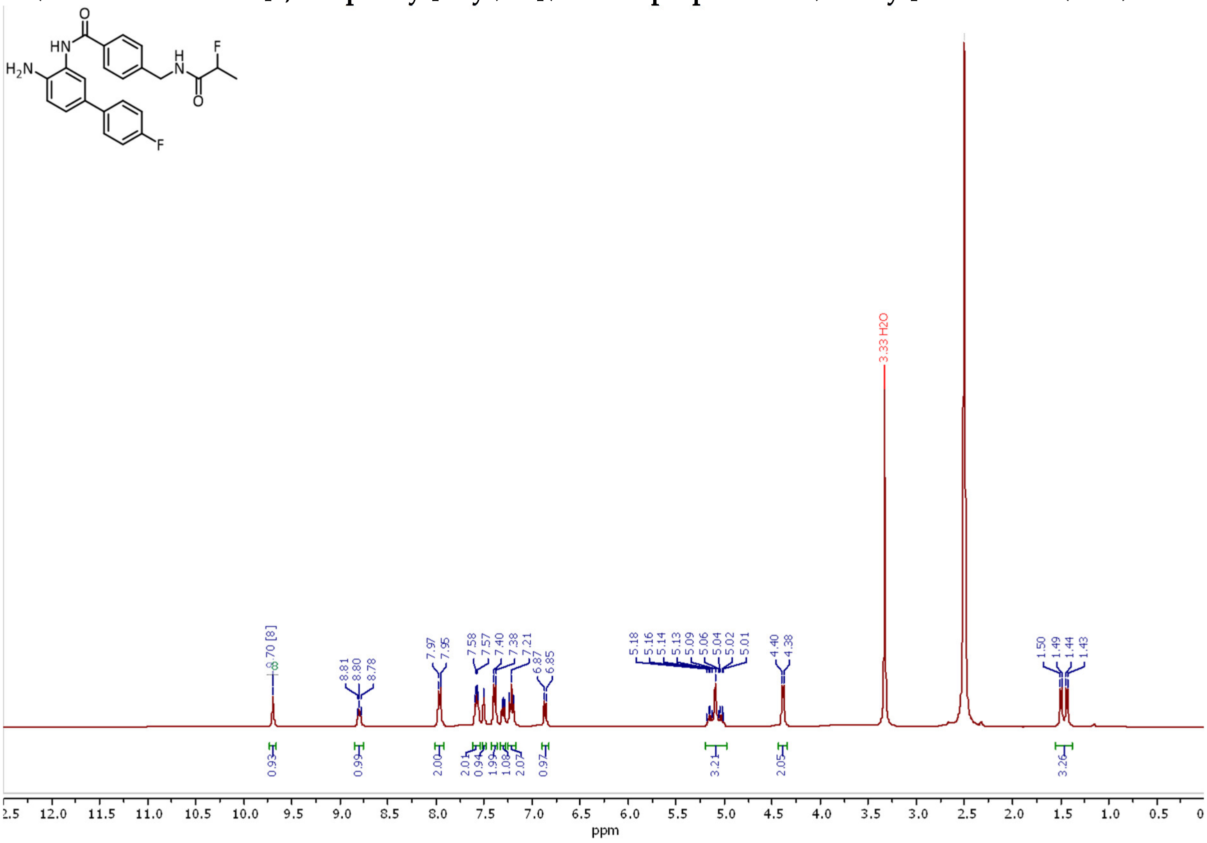

Figure S23. ^{1}H-NMR of **BA5**

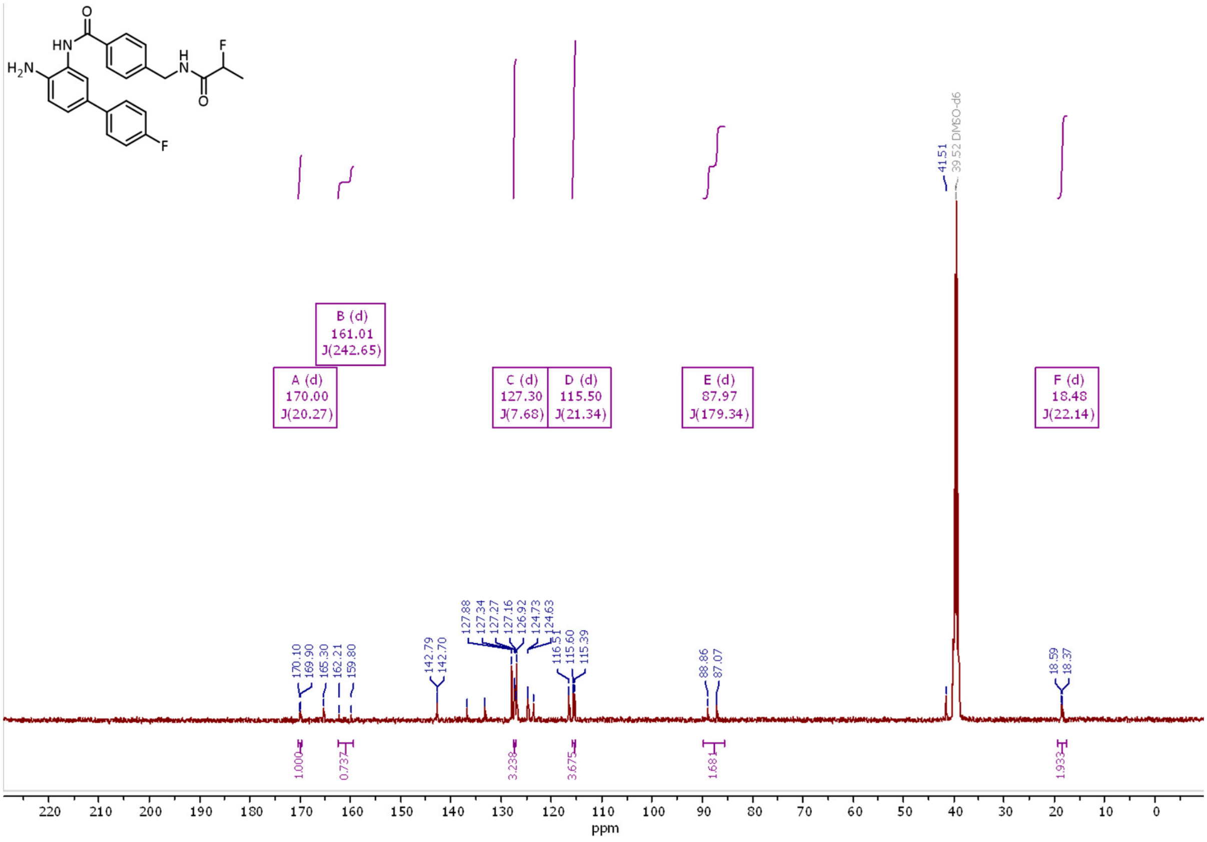

Figure S24. ^{13}C-NMR of **BA5**

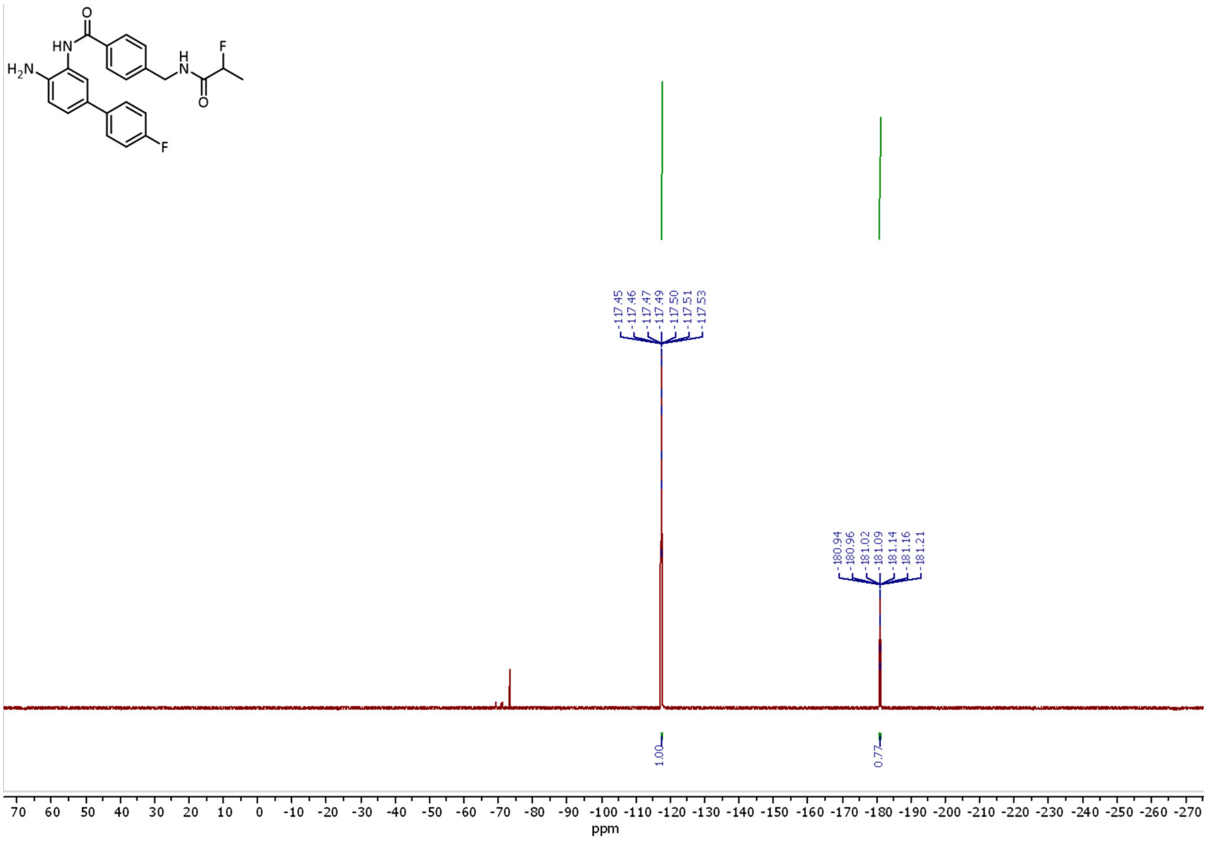

Figure S25. ^{19}F-NMR of **BA5**

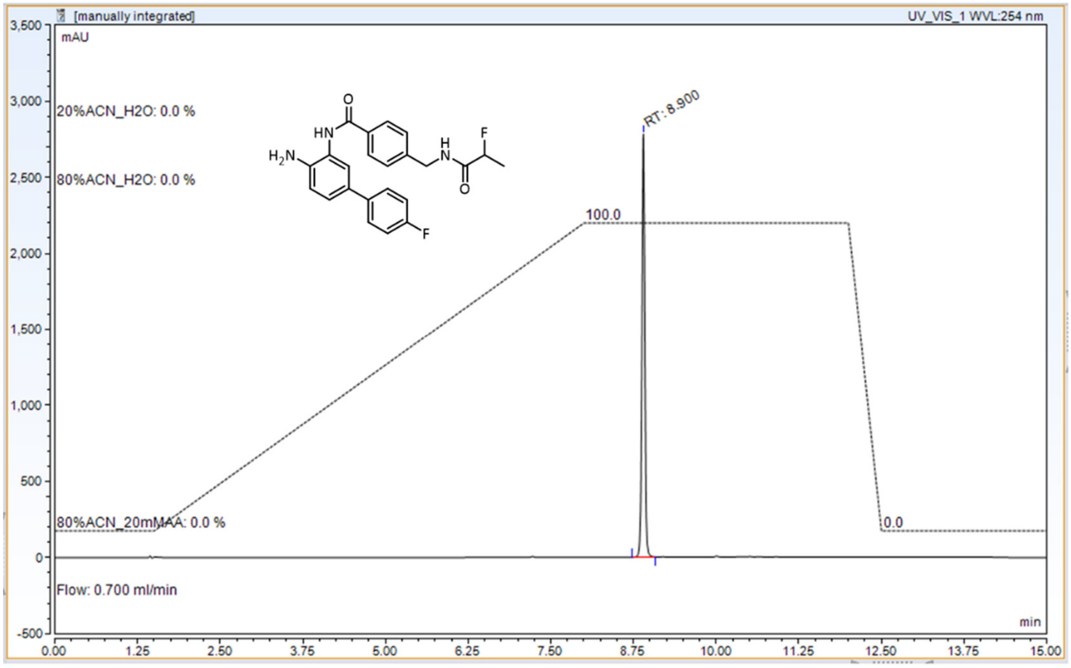

Figure S26. LC-MS chromatogram of **BA5**

N-(4-amino-4'-fluoro-[1,1'-biphenyl]-3-yl)-4-(2-fluoropropanamido)benzamide (BA6)

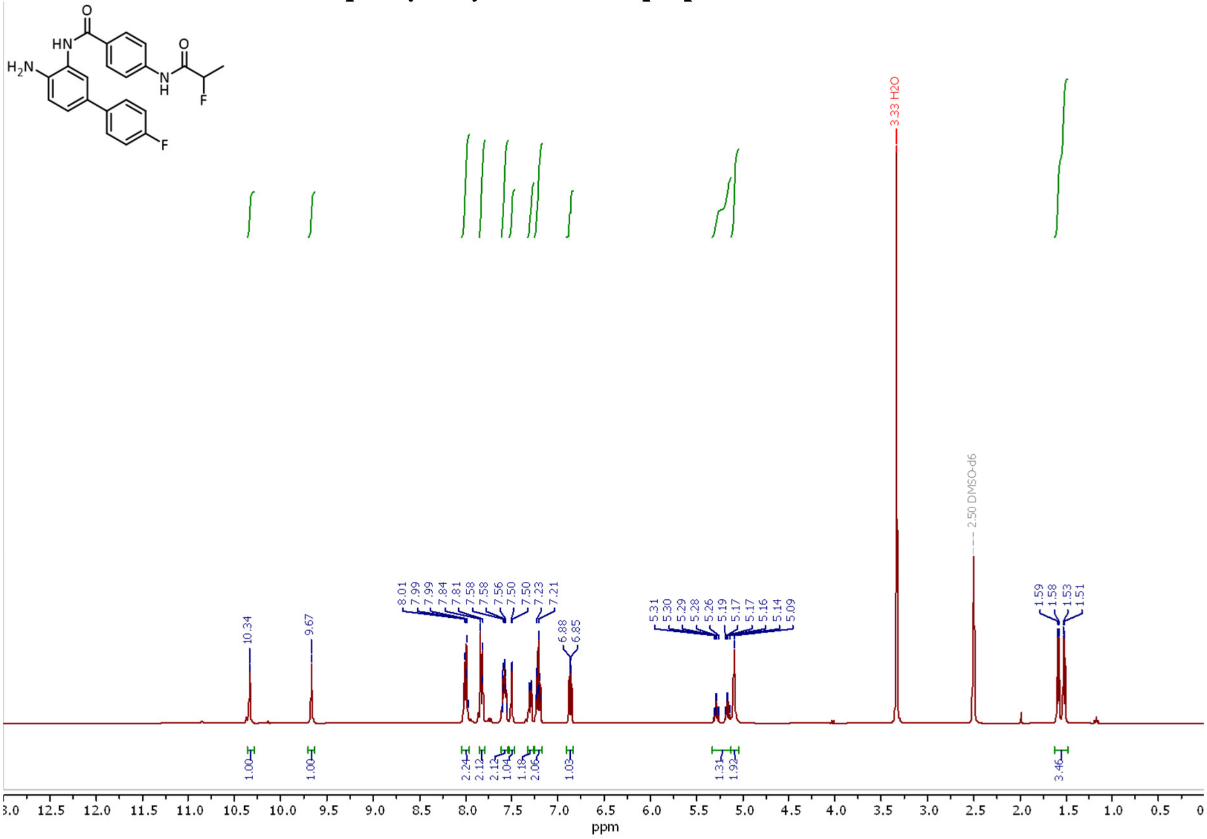

Figure S27. ^{1}H-NMR of **BA6**

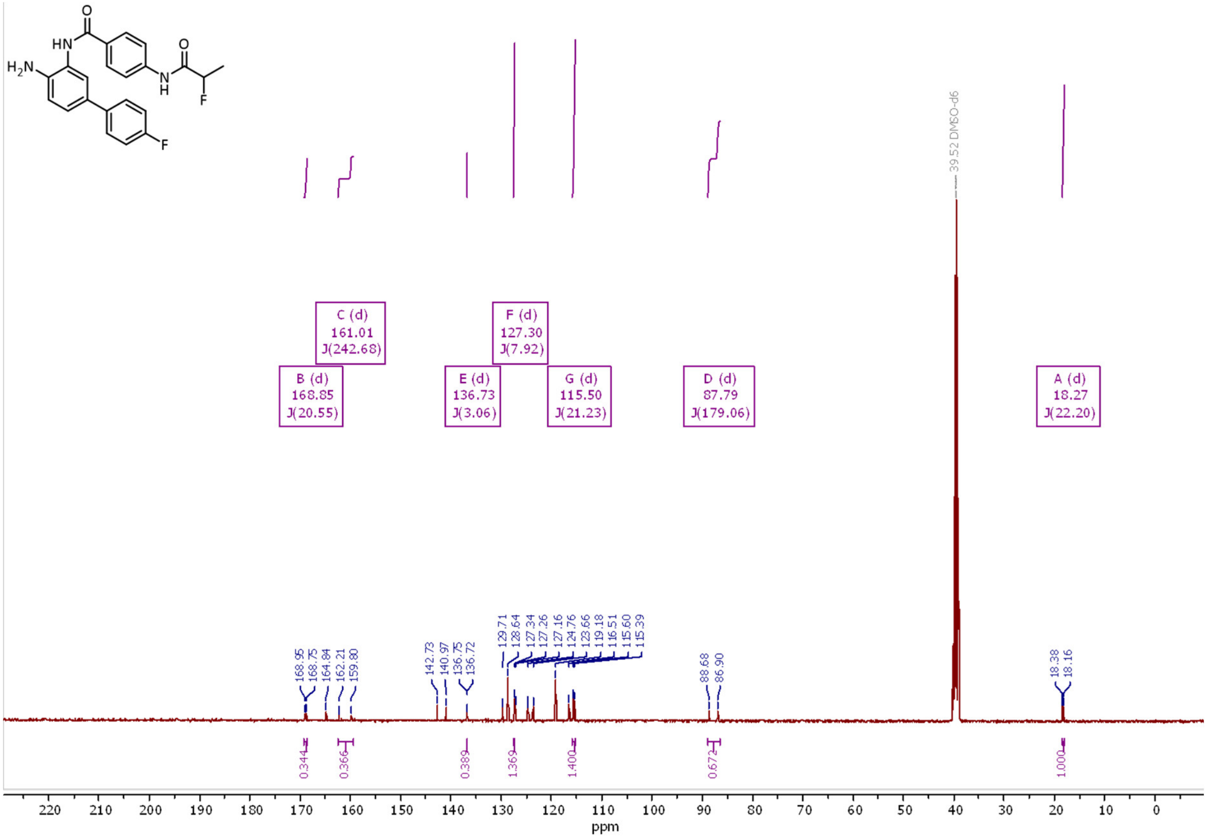

Figure S28. ^{13}C-NMR of **BA6**

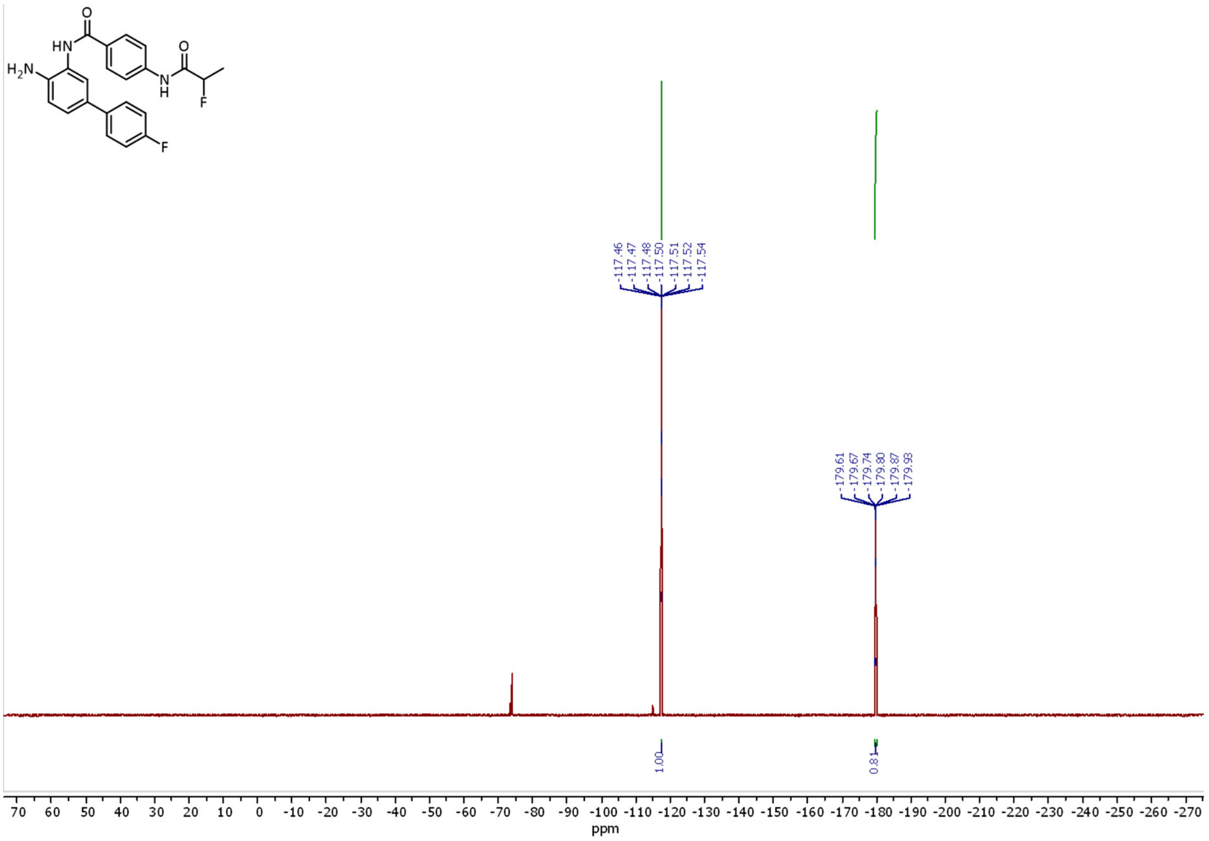

Figure S29. ^{19}F-NMR of **BA6**

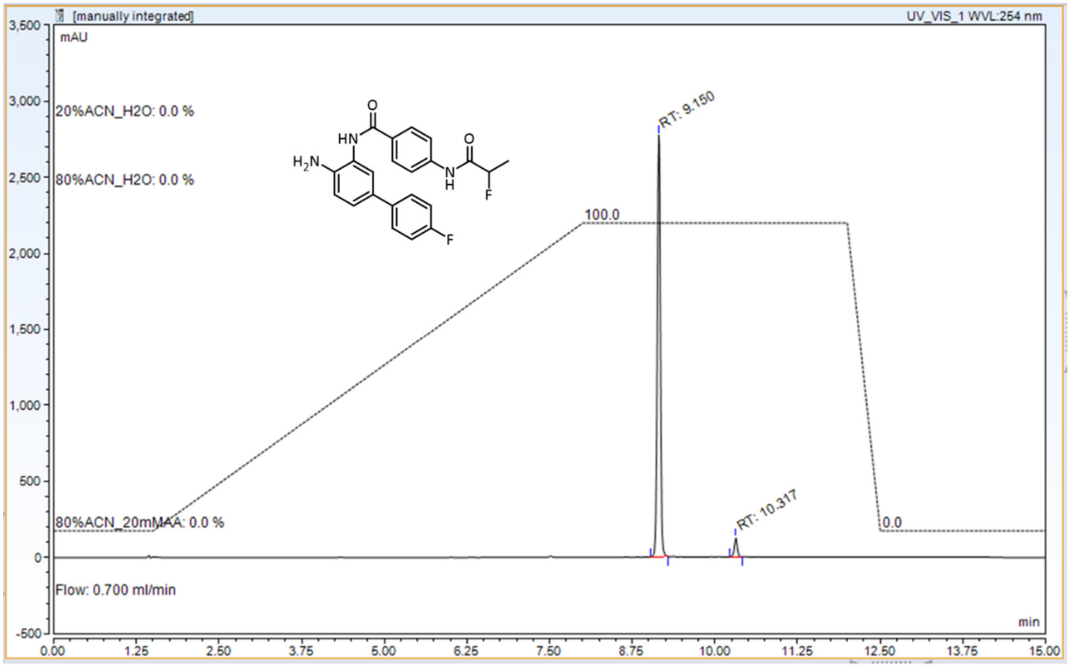

Figure S30. LC-MS chromatogram of **BA6**

N-[2-amino-5-(furan-2-yl)phenyl]-4-[(2-fluoropropanamido)methyl]benzamide (BA7)

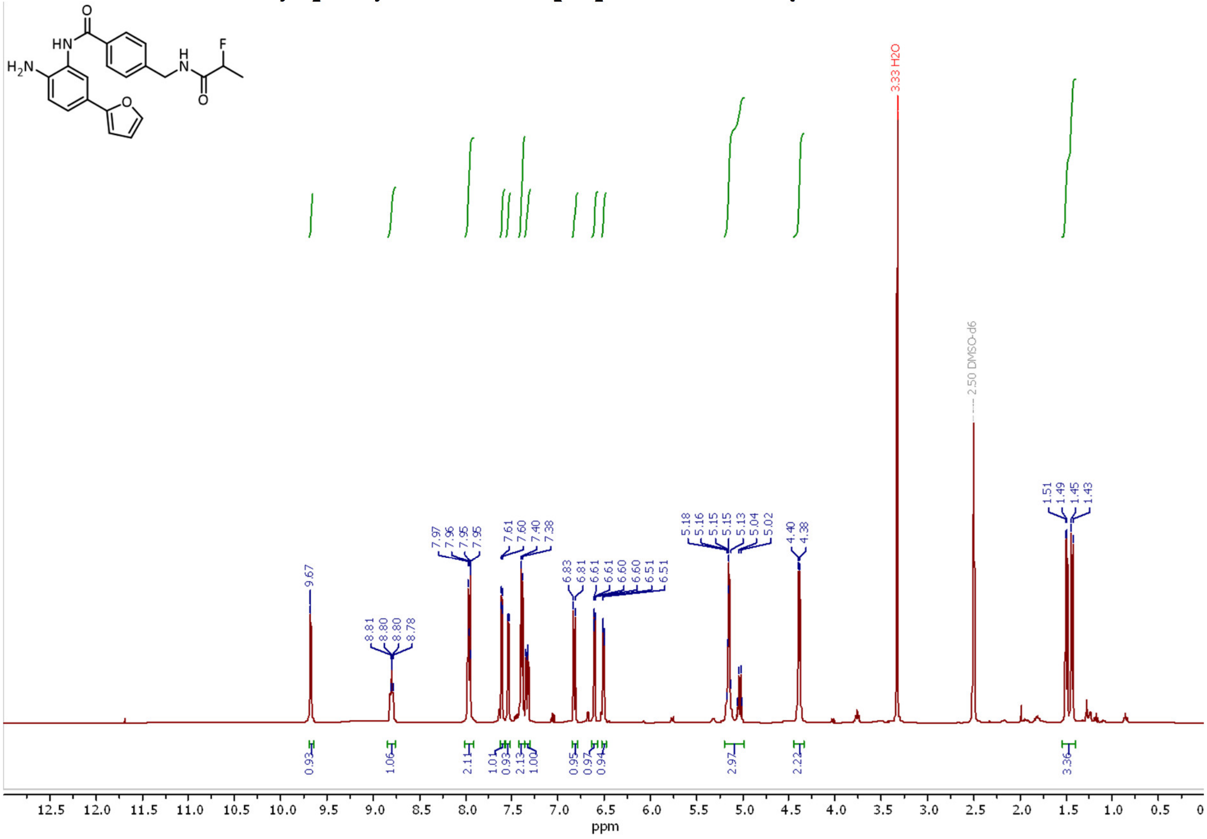

Figure S31. ^{1}H-NMR of **BA7**

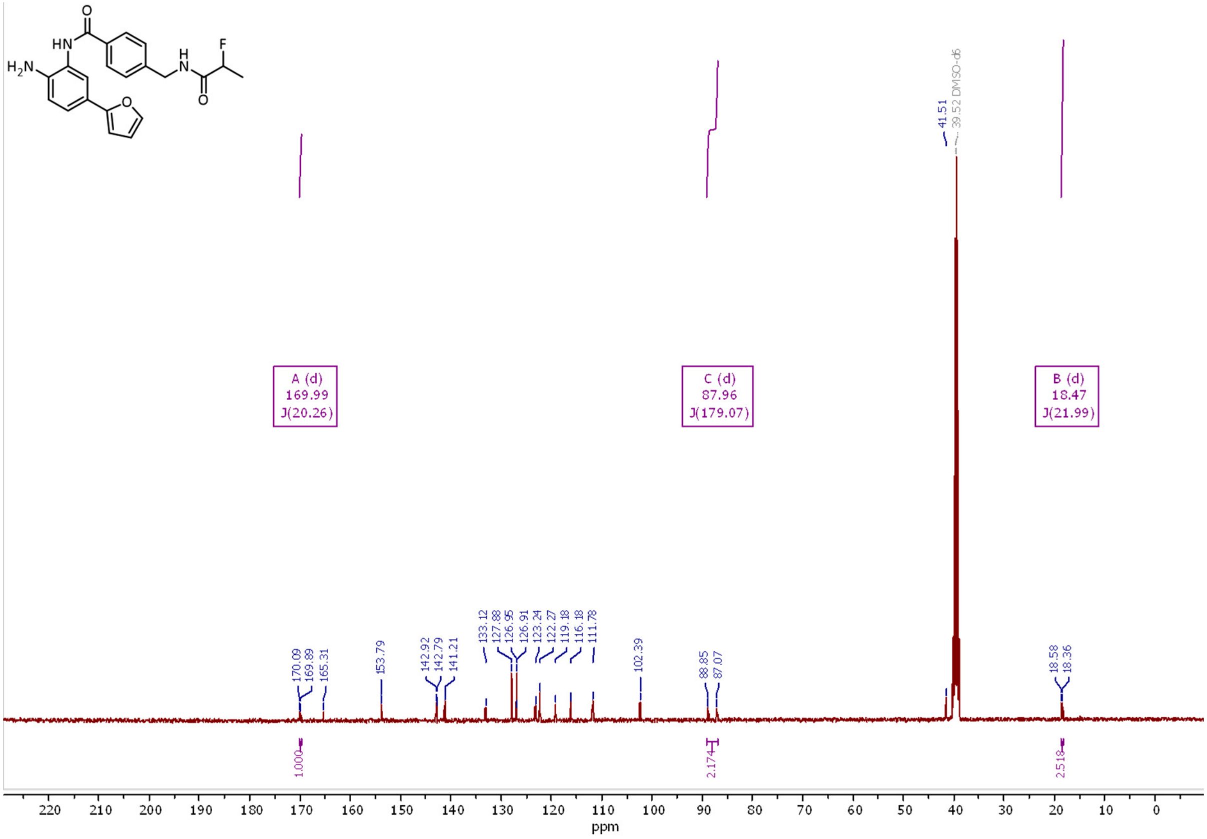

Figure S32. ^{13}C-NMR of **BA7**

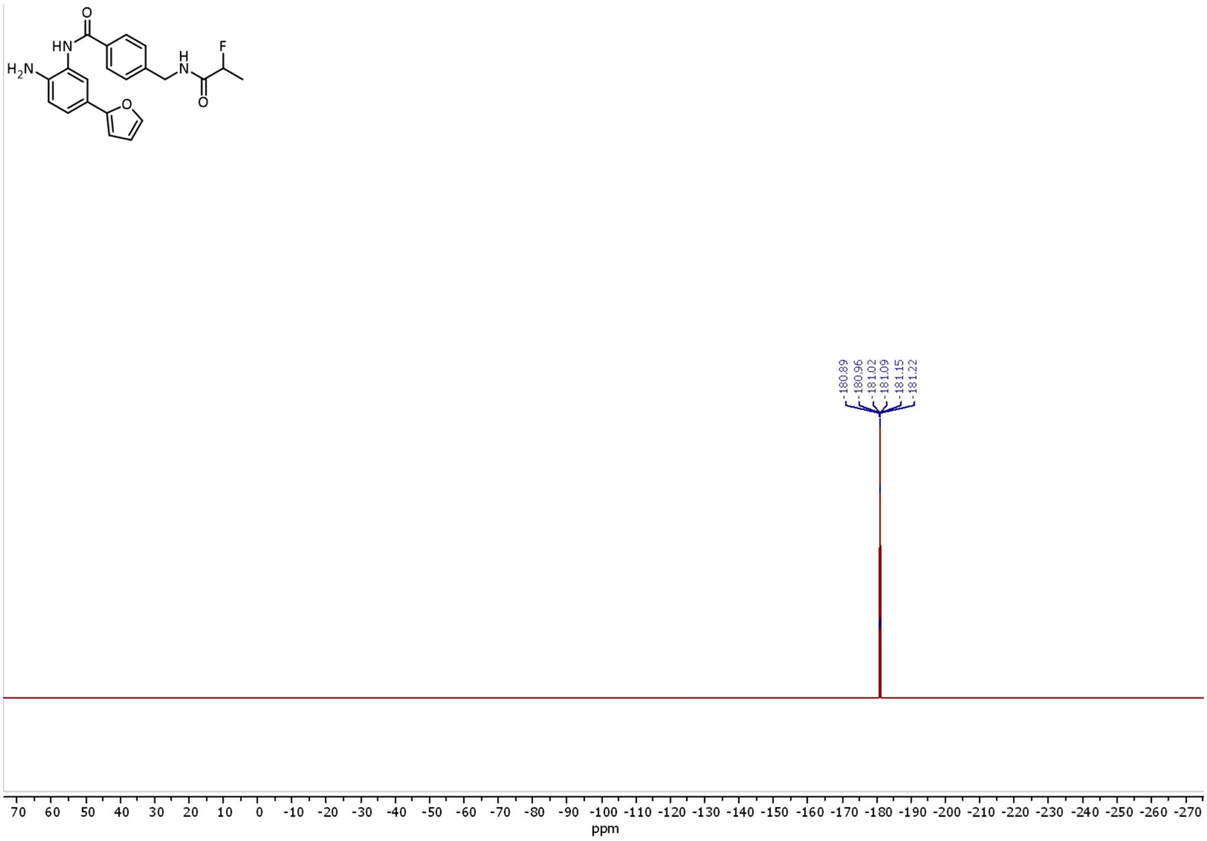

Figure S33. ^{19}F-NMR of **BA7**

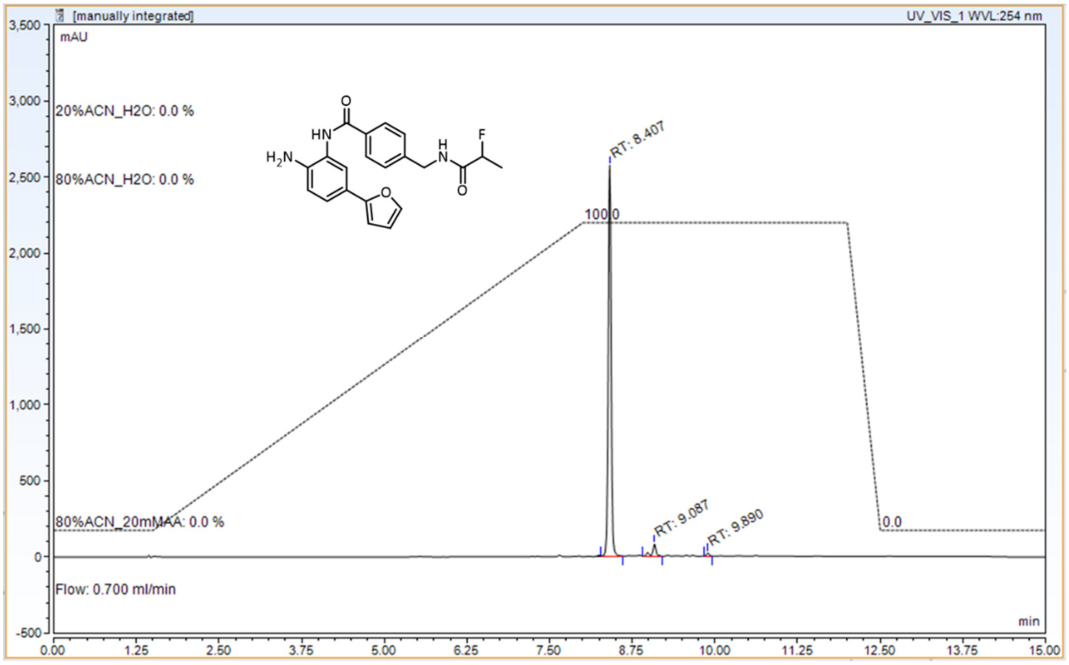

Figure S34. LC-MS chromatogram of **BA7**

***N*-[2-amino-5-(furan-2-yl)phenyl]-4-(2-fluoropropanamido)benzamide (BA8)**

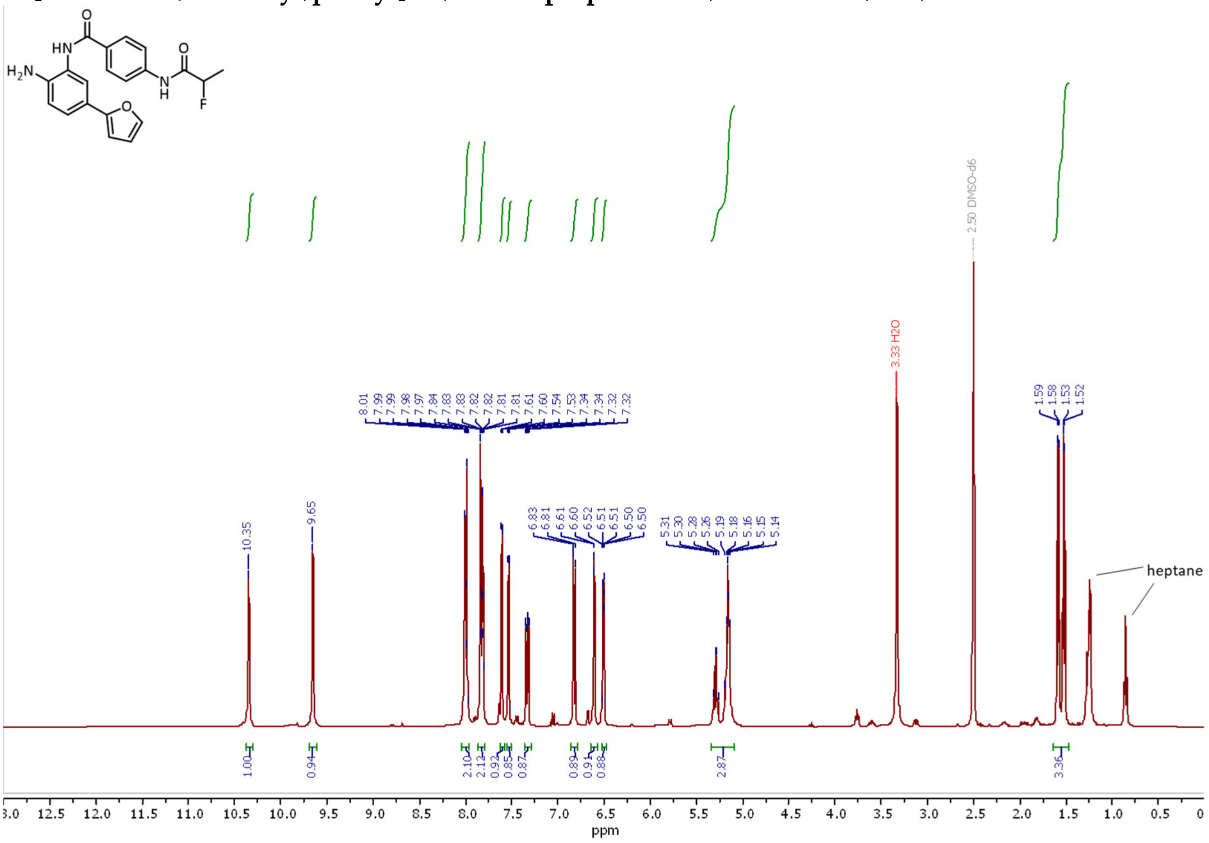

Figure S35. ^{1}H-NMR of **BA8**

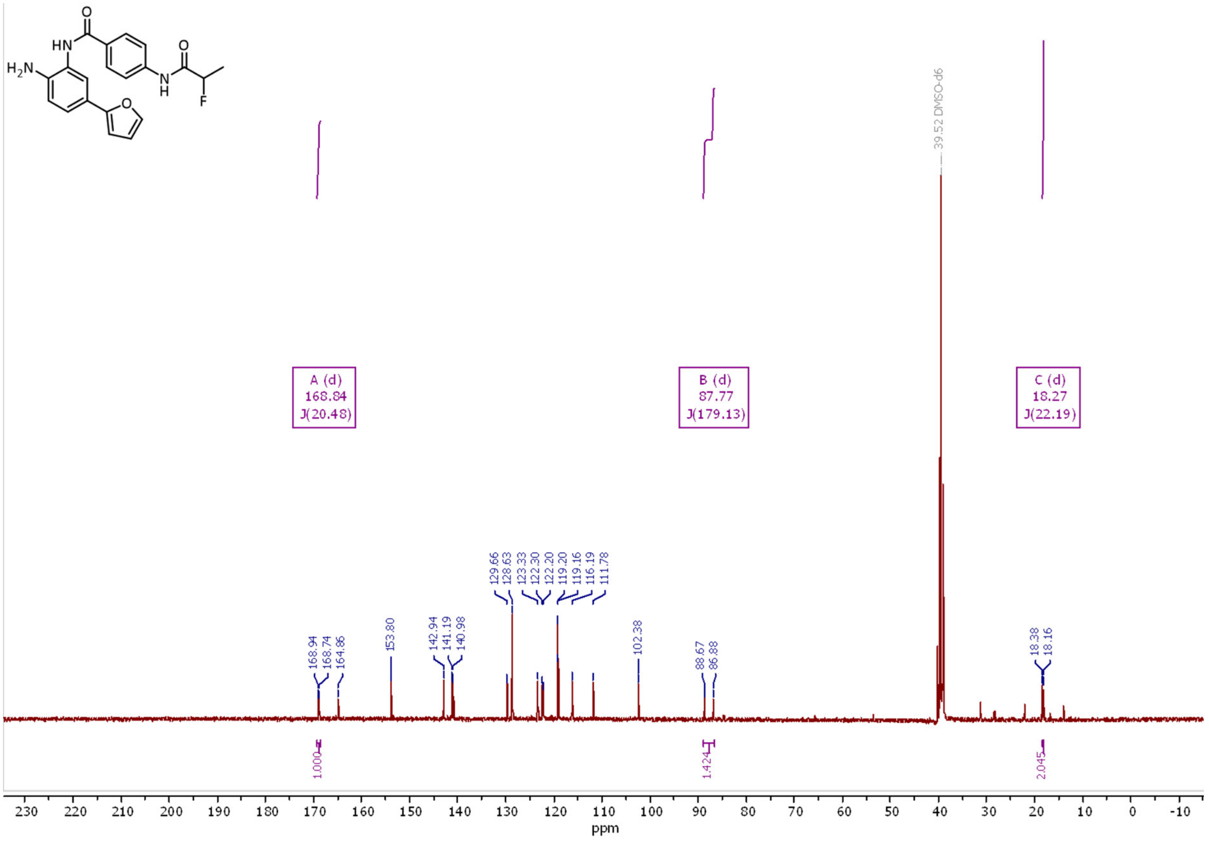

Figure S36. ^{13}C-NMR of **BA8**

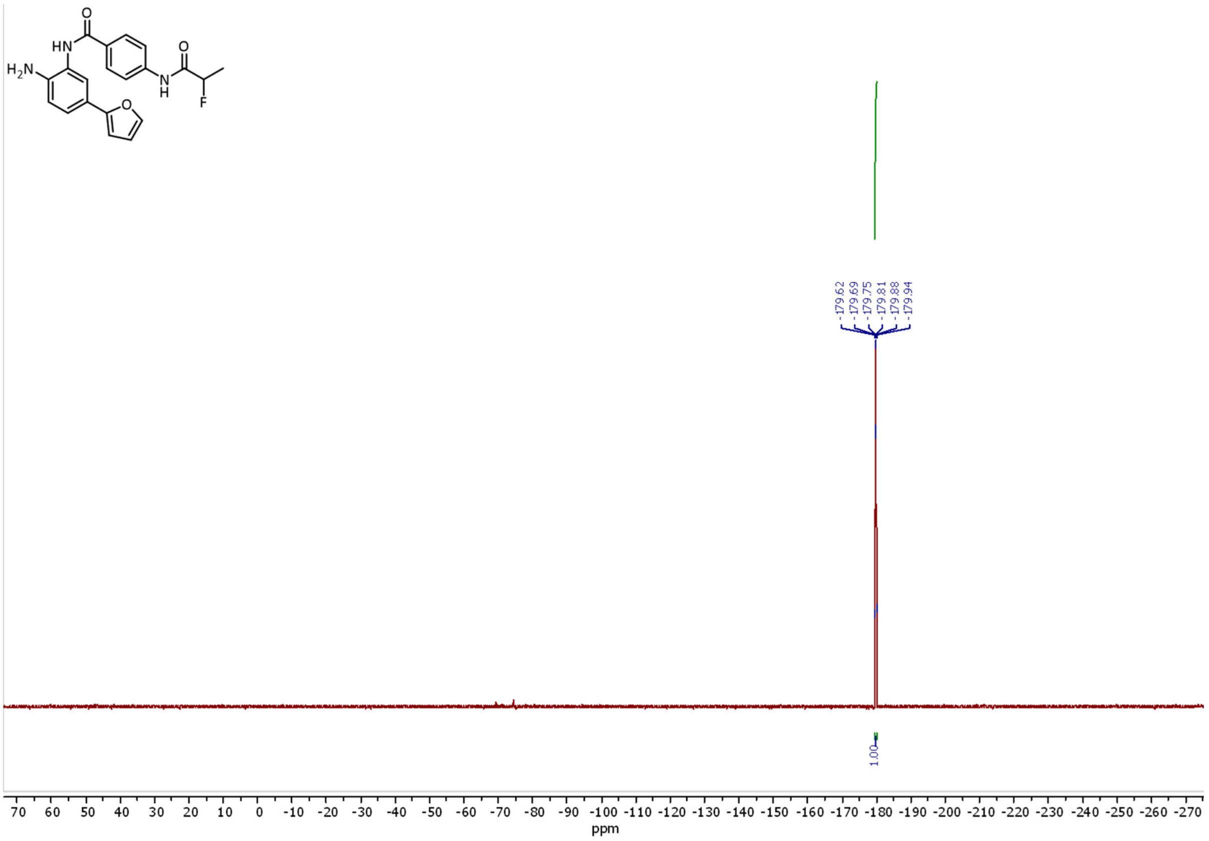

Figure S37. ^{19}F-NMR of **BA8**

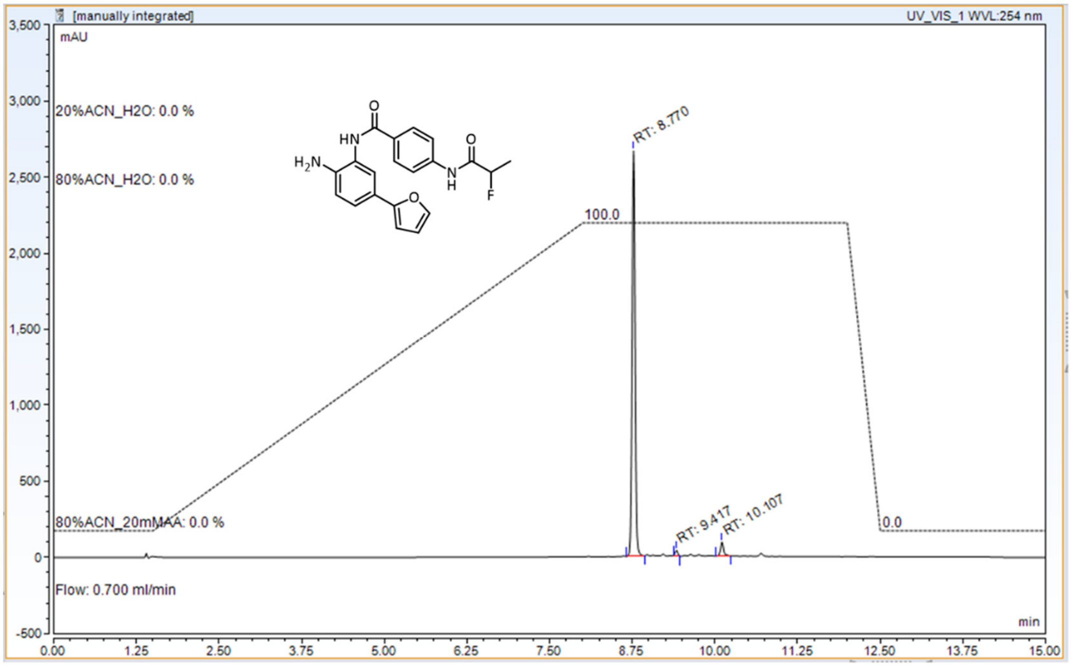

Figure S38. LC-MS chromatogram of **BA8**

N-[2-amino-5-(furan-3-yl)phenyl]-4-[(2-fluoropropanamido)methyl]benzamide (BA9)

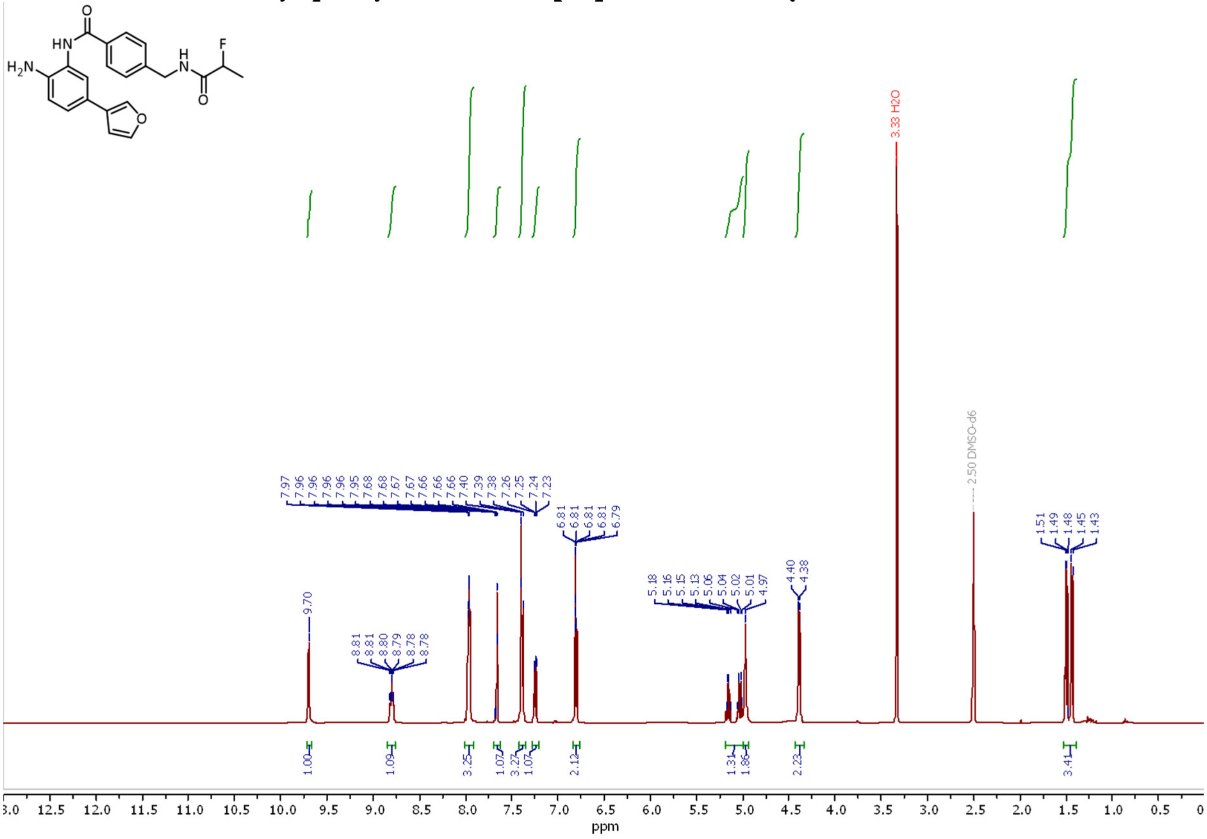

Figure S39. ¹H-NMR of **BA9**

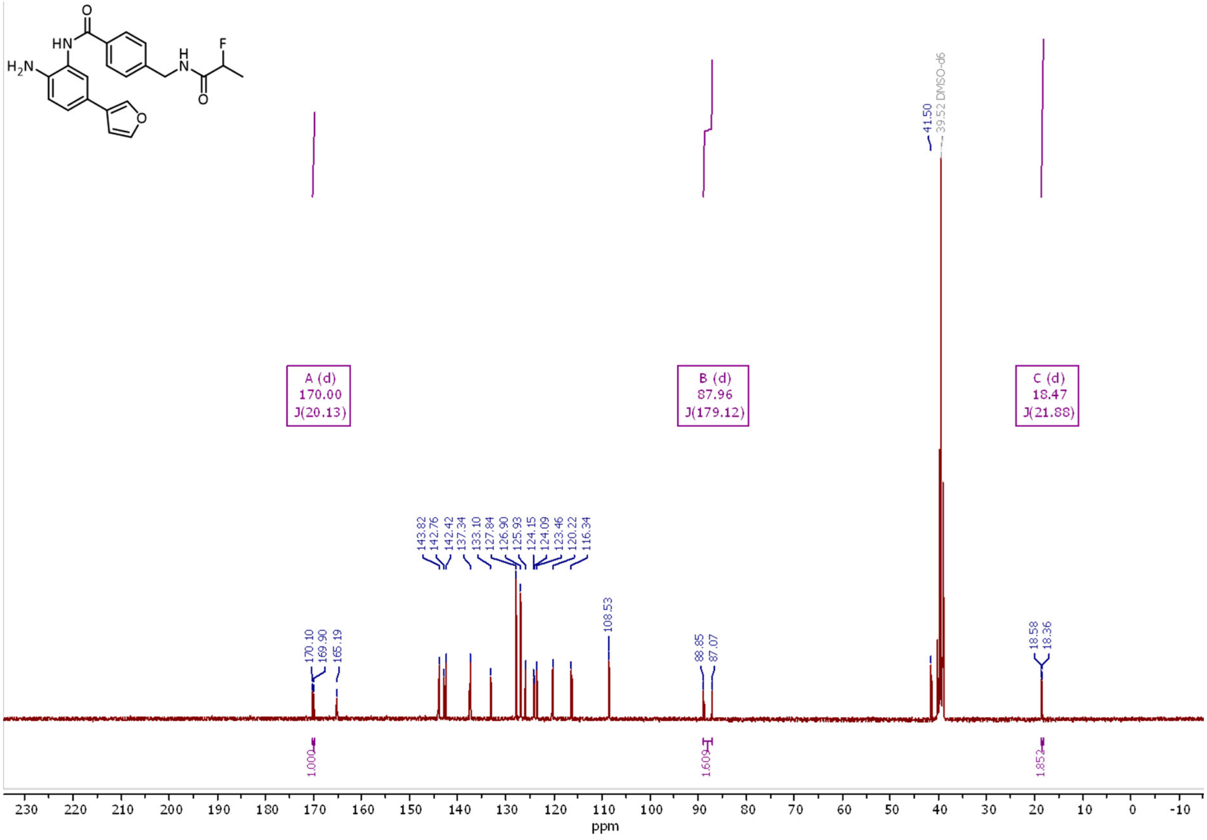

Figure S40. ¹³C-NMR of **BA9**

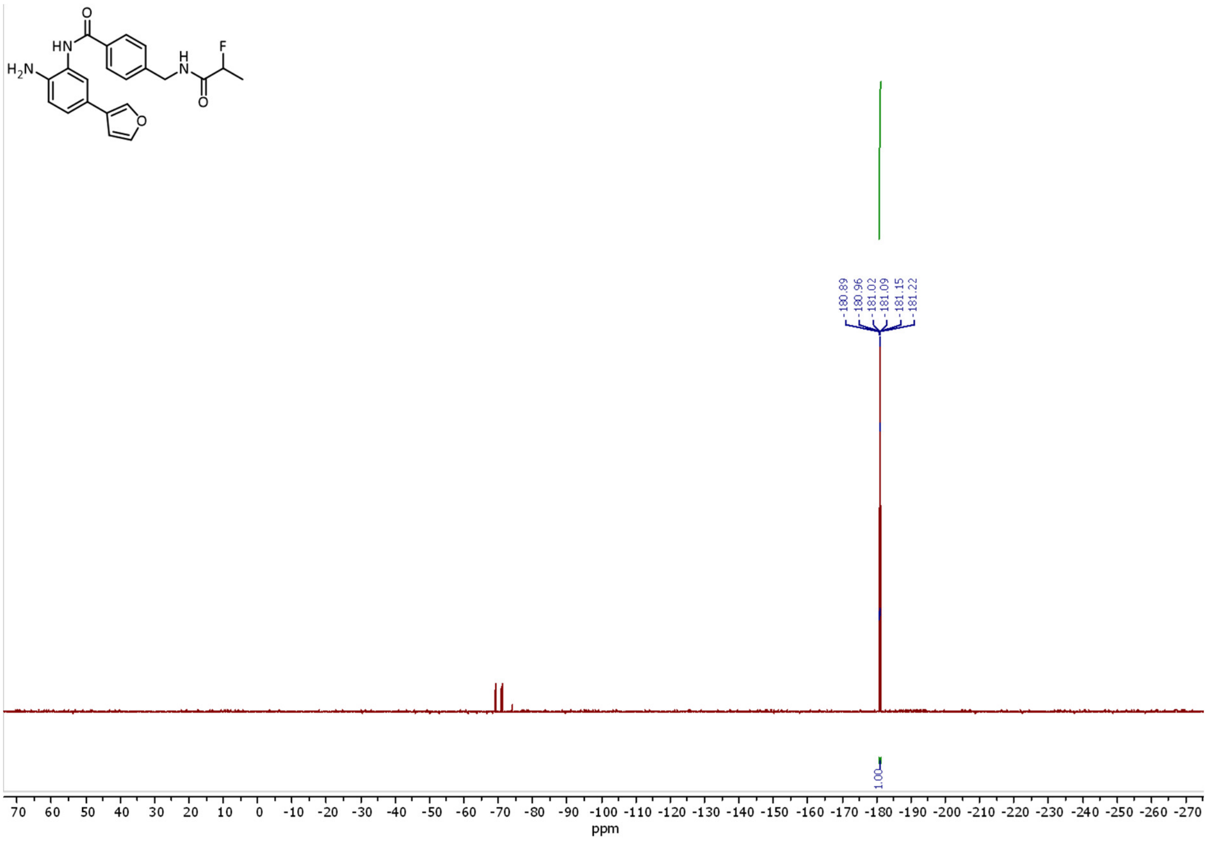

Figure S41. ^{19}F-NMR of **BA9**

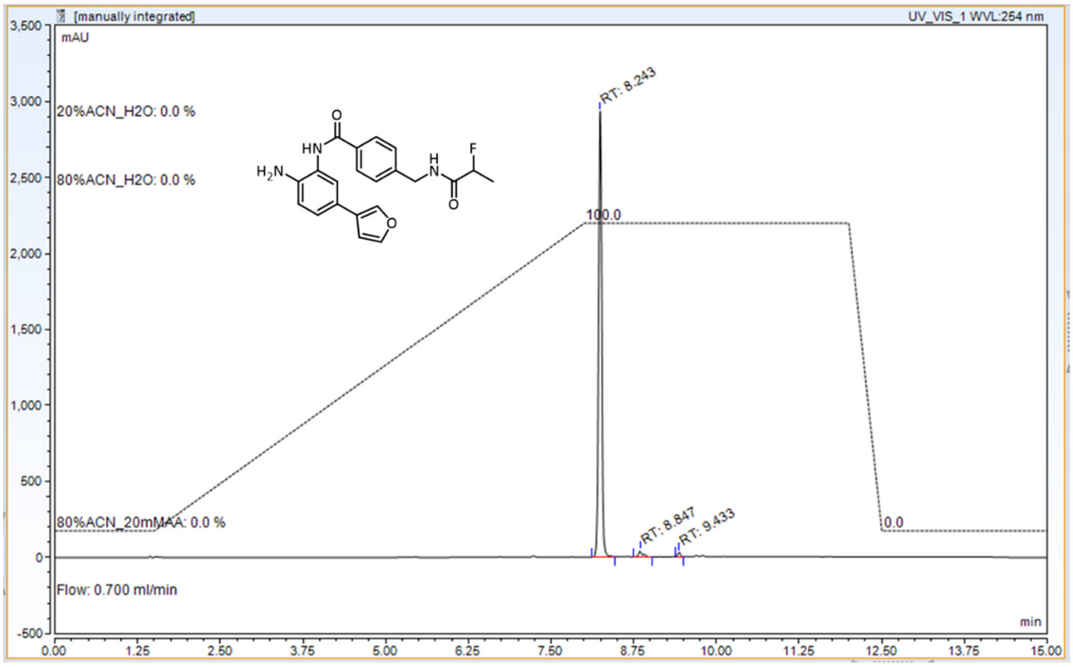

Figure S42. LC-MS chromatogram of **BA9**

N-[2-amino-5-(furan-3-yl)phenyl]-4-(2-fluoropropanamido)benzamide (BA10)

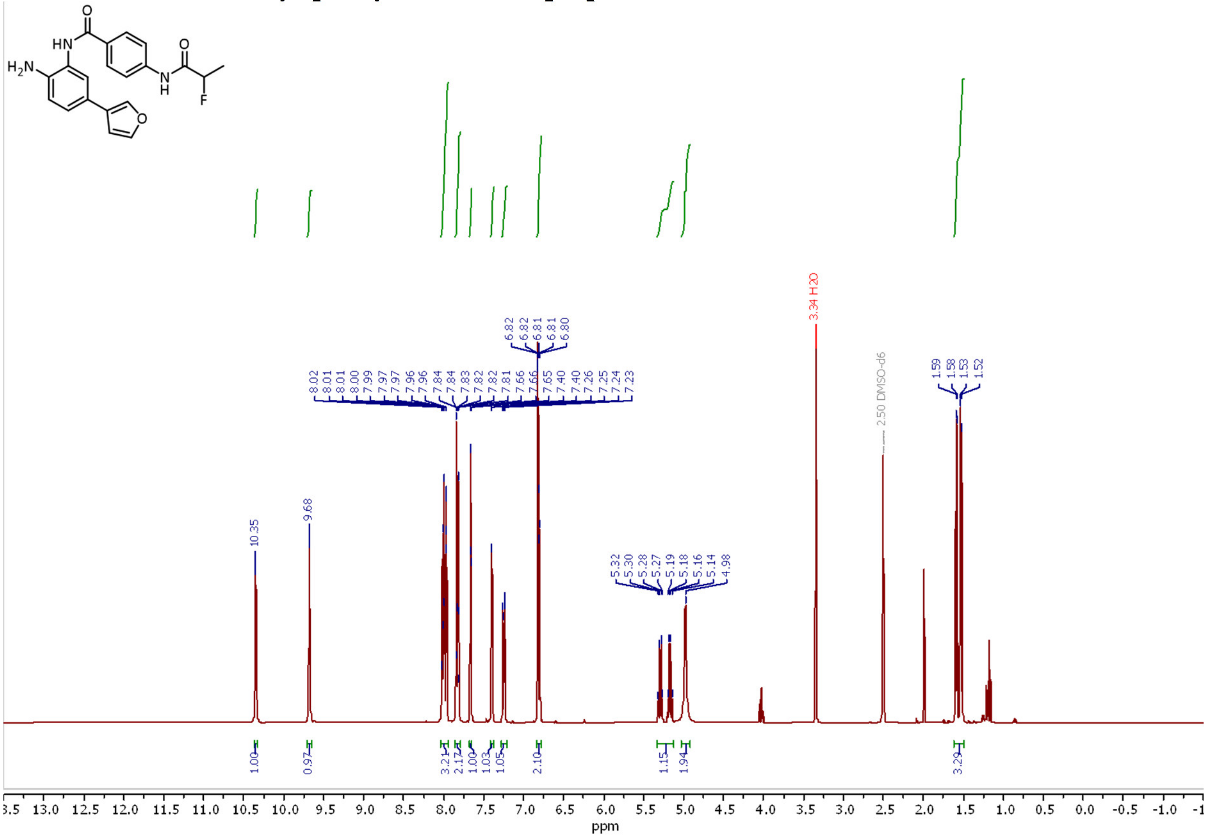

Figure S43. ^{1}H-NMR of **BA10**

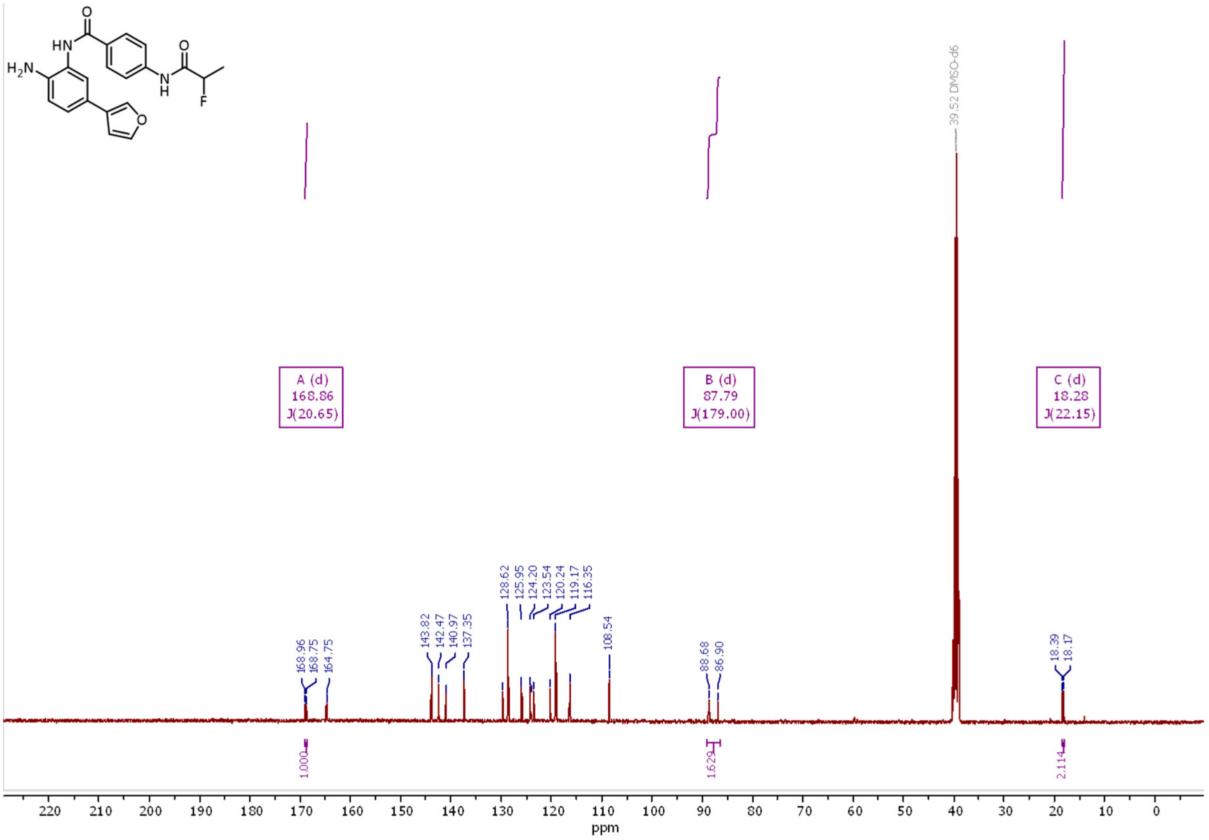

Figure S44. ^{13}C-NMR of **BA10**

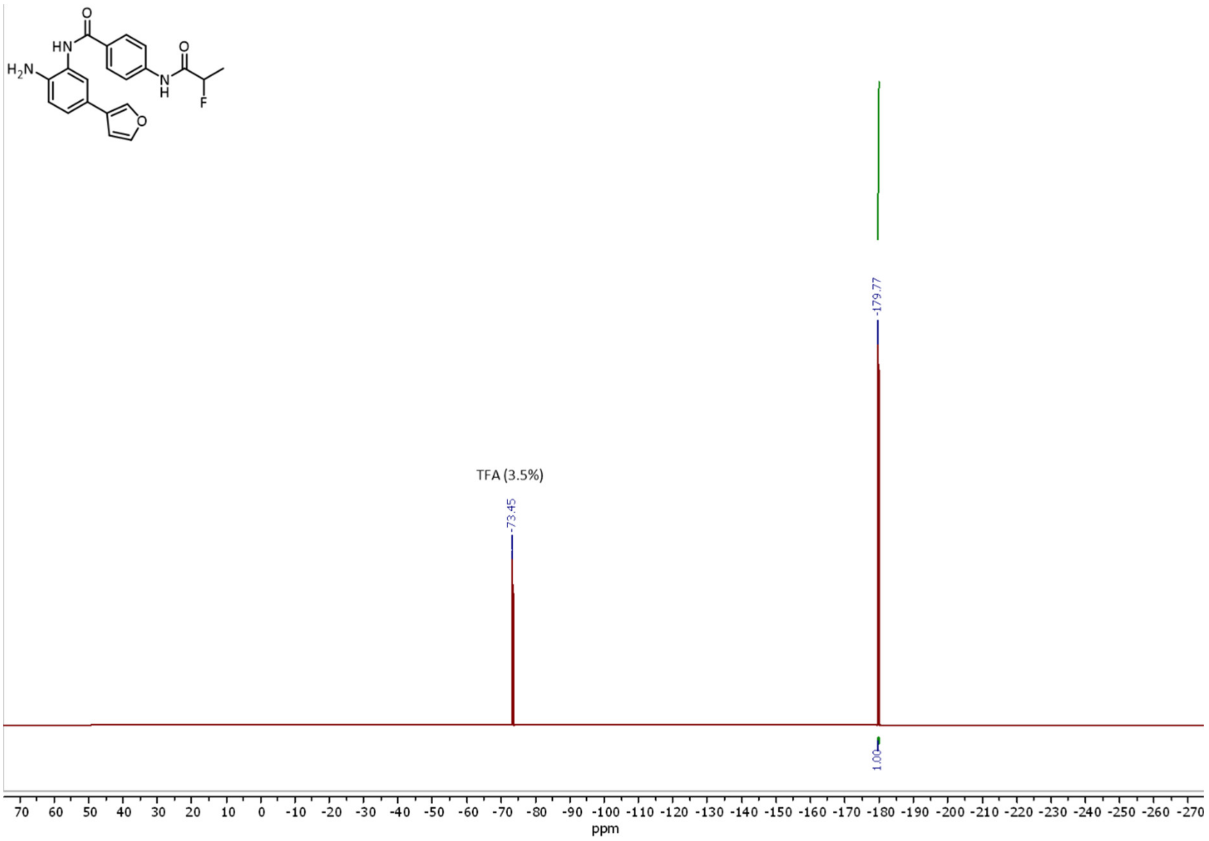

Figure S45. ^{19}F-NMR of **BA10**

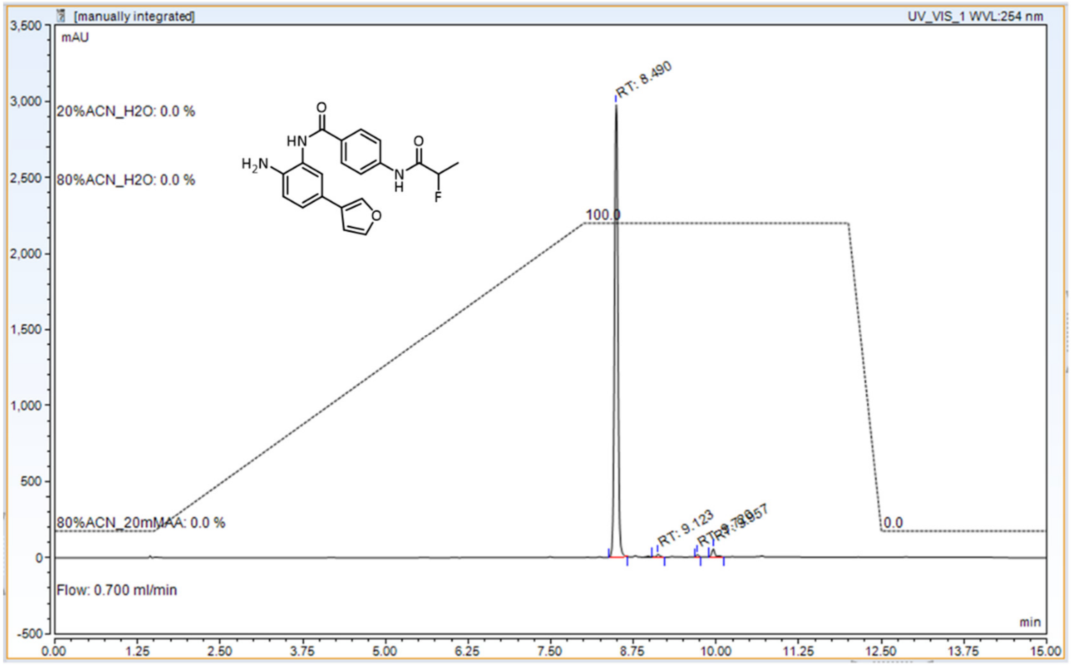

Figure S46. LC-MS chromatogram of **BA10**
